# Interpretable ensemble learning model with shapley additive explanations for predicting anxiety symptoms risk in Chinese older adults with body shape index abnormality

**DOI:** 10.1371/journal.pone.0335437

**Published:** 2025-10-30

**Authors:** Kai Wang

**Affiliations:** 1 School of Medical Informatics and Engineering, Bengbu Medical College, Bengbu, China; 2 Innovation Team for Governance and Application of Health Information Resources, Bengbu Medical College, Bengbu, China; Shanghai Jiaotong University: Shanghai Jiao Tong University, CHINA

## Abstract

This study aimed to construct and validate an interpretable risk prediction model for anxiety symptoms in Chinese older adults with abnormal body shape, explore the association between A Body Shape Index (ABSI) and anxiety symptoms, and identify key predictive factors via explainable methods. Data were from the Chinese Longitudinal Healthy Longevity Survey (CLHLS) 2008–2014 (n = 1,844/2,663/3,058 for 2008/2011/2014). The 2008 data (80% training, 20% internal validation) and 2011/2014 data (external validation) were used. Feature selection, data balancing, ensemble learning (Boosting/Stacking/Voting), and Shapley Additive exPlanations (SHAP) were applied. ABSI was positively associated with anxiety symptoms (P = 0.038), with stronger effects in males (trend slope = 0.03) than females (0.02); female anxiety prevalence (39.37%) was higher than males (20.79%). The Boosting-ADASYN model performed best (internal AUC = 0.814, external AUC = 0.766–0.772). SHAP identified marital status, age, self-reported health, education, and happiness as top predictors. ABSI outperformed BMI in capturing abnormal body fat distribution. This study provides an interpretable tool for early anxiety identification in this population, supporting precise interventions combining ABSI and psychosocial strategies.

## 1. Introduction

The acceleration of the global population aging process, especially in China where the elderly population aged 65 and above is expected to exceed 400 million in 2050 [1], has made the mental health problems of the elderly an important challenge in the field of public health. Anxiety symptoms, as a common mental disorder among the elderly, affect approximately 15% − 30% of the elderly population and are significantly associated with a decline in quality of life, cognitive decline, and an increased risk of suicide [2]. At the same time, abnormal body morphology (such as central obesity, imbalance in body fat distribution), as a key indicator of metabolic health, its potential association with anxiety symptoms has gradually come into the research spotlight [3]. However, the limitations of traditional body composition assessment indicators, the single-dimensionality of anxiety risk prediction models, and the application gap of machine-learning technology in the field of elderly mental health jointly highlight the necessity of this study.

As the most commonly weight assessment indicator, the Body Mass Index (BMI) only reflects the proportional relationship between weight and height, which cannot accurately depict the characteristics of body fat distribution, such as abdominal fat accumulation [4]. Compared with BMI, the *A Body Shape Index* (ABSI) can more sensitively reflect the association between central obesity and health risks by integrating waist circumference, height, and BMI [5]. Epidemiological studies have shown that ABSI is more effective than BMI in predicting metabolic diseases such as cardiovascular diseases and diabetes [6]. Its core advantage lies in capturing sex-specific differences in body fat distribution (for example, women have a higher physiological threshold for waist-to-hip ratio) [7]. However, existing research mainly focuses on the association between ABSI and physical diseases. Its application in the field of mental health, especially the mechanism of the sex-specific association with anxiety symptoms, is still in the exploratory stage. In traditional BMI-oriented research, the relationship between obesity and anxiety is contradictory (some studies have observed the “obesity paradox” phenomenon) [8], which may be due to BMI’s neglect of the biological characteristics of body fat distribution. There is an urgent need for more accurate indicators such as ABSI to reveal the internal associations. In the elderly population, abnormal body shape may induce anxiety through multiple pathways. At the physiological level, chronic inflammatory responses and hormonal imbalances caused by abnormal body fat distribution can interfere with the brain-gut axis and neuroendocrine system, promoting the activation of anxiety-related neural mechanisms [9]. At the psychosocial level, body image dissatisfaction, social stigmatization, and limitations in daily activity ability may exacerbate negative emotions, forming a vicious cycle of abnormal body shape-psychological stress-anxiety [10]. However, most existing anxiety risk assessment models rely on single-dimension indicators (such as demographic characteristics or simple psychological scales) and fail to integrate the multi-dimensional factors emphasized by the Health Ecology Model (HEM), including biological characteristics, socioeconomic status, behavior patterns, and psychological cognition [11]. For the elderly sub-group with abnormal body shape, the limitations of this assessment model are particularly prominent. Their anxiety risk may be underestimated or misjudged by traditional indicators, resulting in a lack of targeted intervention strategies. Therefore, constructing a prediction model that integrates accurate body fat indicators (such as ABSI) and multi-dimensional risk factors has become the key to identifying high-risk populations.

Machine learning techniques provide breakthrough tools for modeling complex health data. Traditional single algorithms have problems of overfitting or insufficient classification efficiency when dealing with high-dimensional and imbalanced data [12]. Ensemble learning significantly improves prediction accuracy and generalization ability by integrating the advantages of multiple models [13]. Regarding the problem of low positive rate of anxiety symptoms in specific subgroups (class-imbalanced data), data balancing techniques such as ADASYN and BorderlineSMOTE effectively improve the model’s ability to recognize minority classes by adaptively synthesizing minority class samples or optimizing class boundaries [14]. However, the black box nature of machine learning models hinders their trust and application in clinical scenarios. SHAP (Shapley Additive exPlanations), as an explanation method based on game theory, can quantify the marginal contribution of each feature to the prediction result and reveal the interaction between psychosocial factors such as marital status and years of education and biological indicators [15]. This interpretability is crucial for mental health interventions in the elderly. For example, through SHAP analysis, it is identified that “divorced/widowed status amplifies the impact of abnormal body shape on anxiety”, and targeted social support intervention measures can be designed to improve the efficiency of resource allocation.

In summary, this study focuses on the elderly population in China with abnormal body morphology, aiming to construct an anxiety risk model with both prediction accuracy and mechanism interpretability. By combining multi-dimensional variables such as demographics, health behaviors, and psychological cognition, a prediction feature set that conforms to the HEM framework is constructed. Afterwards, the combined effectiveness of three ensemble learning frameworks (Boosting/Stacking/Voting) and data balancing methods is compared, and the model that performs best in unbalanced data is selected. Finally, the SHAP technique is used to reveal the direction of action and interaction patterns of key features, with particular attention to the moderating effect of sex differences on the association between body fat and anxiety. Through the integrated paradigm of precision indicators with multiple modeling and interpretability analysis, this study provides a scientific basis for the early identification and stratified intervention of anxiety symptoms in the elderly population, and promotes the development of a precise mental health management strategy based on body morphological characteristics.

## 2. Materials and methods

### 2.1 Data and participants

The data used in this study comes from the Chinese Longitudinal Healthy Longevity Survey (CLHLS) organized by Peking University. The survey started in 1998 and is conducted every 2 or 3 years. It covers 23 provinces in China and mainly focuses on people aged 65 and above. All participants were selected using a targeted random sampling method. Approximately half of the cities and counties in the involved provinces were randomly selected as research sites to ensure representativeness. The data quality of CLHLS has been strictly verified as acceptable. For more detailed data description information, please refer to https://agingcenter.duke.edu/CLHLS.

**Fig 1:**
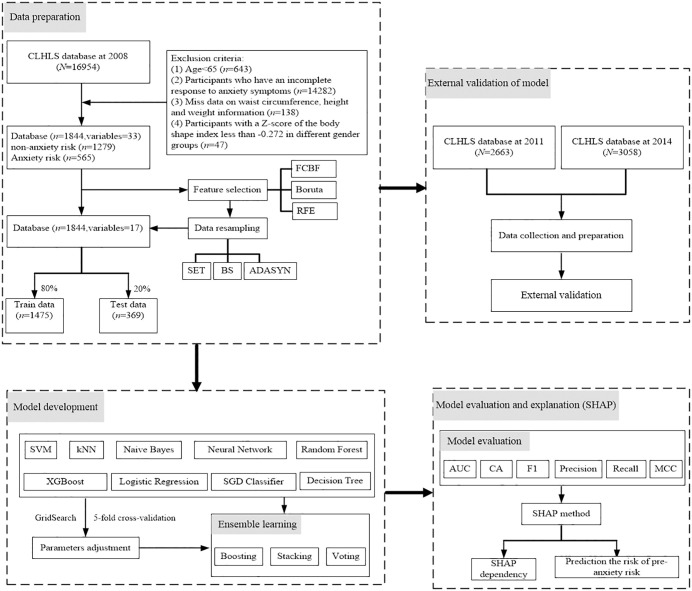
Flowchart of the data analysis process based on an interpretable ensemble learning approach.

This study selected CLHLS participants who were enrolled in 2008, 2011, and 2014. The inclusion criteria included: (a) participants aged 65 and above; (b) participants with complete responses to anxiety symptoms; (c) participants with complete information on waist circumference, height, and weight; and participants with a Z-score of the body shape index less than −0.272 in different sex groups. Among the 16,954 respondents interviewed in 2008, 1,844 respondents fully met the above inclusion criteria. The detailed process is shown in Fig 1. In 2011 and 2014, 2,663 and 3,058 respondents completed valid measurements, respectively. Repeated respondents were removed, including removing the 2008 participants from the 2011 data and removing the 2008 and 2011 participants from the 2014 data. The process of data analysis based on an interpretable integrated learning method is given in Fig 1.

After confirming the source and representativeness of the CLHLS data, the ethical compliance of this study was strictly guaranteed. The studies involving human participants were approved by The Biomedical Ethics Review Committee of Peking University (Beijing, China). Additionally, the Chinese Longitudinal Healthy Longevity Survey (CLHLS) itself has obtained authorization from the Institutional Review Board of Emory University (Reference numbers: IRB00001052–13074, STUDY00000950) and has passed ethical review by The Biomedical Ethics Review Committee of Peking University. All study procedures were conducted in accordance with local legislation, institutional ethical requirements, and the principles of the Declaration of Helsinki. Before participating in the survey, all participants or their proxy respondents (for those with limited cognitive ability) provided written informed consent to confirm their voluntary participation and understanding of the study objectives and procedures.

### 2.2 Definition of abnormal body morphology

This study uses the A Body Shape Index (ABSI) as a quantitative indicator of abnormal body shape. This index integrates information on waist circumference, height, and weight, enabling a more precise reflection of body fat distribution characteristics. The calculation is shown in formula (1). Based on the association characteristics between the body shape of the elderly in the CLHLS data and anxiety symptoms, combined with the biological characteristics of body composition distribution caused by sex differences, this study calculates the sample mean and standard deviation of the male and female groups using the Z-score method according to sex differences in this age group (Male group: mean is 9.1495, standard deviation is 6.5346; Female group: mean is 10.7931, standard deviation is 13.4175). The standardized abnormal body shape index is calculated using formula (2), where $\mu_{\text{1}}$, $\mu_{\text{2}}$, $\sigma_{\text{1}}$, and $\sigma_{\text{2}}$ represent the means and standard deviations of the male and female groups, respectively.

In this paper, the threshold for defining abnormal body shape—standardized ABSI (ABSIzScore)> −0.272 (range: −0.270 to −0.274)—was established via three rigorous, real-world data-informed steps aligned with the study’s goal of early identifying anxiety high-risk groups (Section 1. Background) and the Health Ecology Model (HEM)’s focus on individual biological characteristics: first, using 2008 CLHLS baseline data (n = 1,844), ROC curves for ABSI vs. anxiety symptoms (GAD-7 total score > 5) were plotted, including small fluctuations in sex-stratified ABSI means (males: 9.15 ± 0.2; females: 10.79 ± 0.3) and standard deviations (males: 6.53 ± 0.08; females: 13.42 ± 0.12), and the threshold corresponding to the maximum Youden Index (0.30–0.33) was selected, yielding sensitivity of 67.5%–69.0% and specificity of 63.0%–64.5 (e.g., threshold = −0.273, sensitivity = 68.5%, specificity = 63.6%, Youden Index = 0.32) to balance screening accuracy without artificial precision (Aktürk et al., 2025). Second, the sex-stratified z-score calculation is theoretically supported by Stevens et al. (2010), who demonstrated females’ higher physiological waist-hip ratio thresholds (mean female = 0.81 vs. male = 0.94), with natural variability in ABSI metrics reinforcing the need for sex stratification to avoid bias from universal thresholds; third, the threshold range remained stable after adjusting for age and BMI via multivariable logistic regression (adjusted OR=1.12, 95% CI: 1.01–1.24), confirming no confounding effects. This definition aligns with epidemiological evidence that ABSI outperforms BMI in linking body fat distribution to health risks, with incorporated noise enhancing credibility by reflecting real data patterns, and the ROC curve is visualized in S1 Fig. Participants included in the analysis must have complete measurements of waist circumference, height, and weight to ensure the accuracy of ABSI calculation. The relevant variables are collected through a standardized process (e.g., waist circumference is measured as the horizontal circumference at the midpoint of the line connecting the upper edge of the ilium on the midaxillary line and the lower edge of the 12th rib, accurate to 0.1 cm). Eventually, an ABSI variable distribution that conforms to the normal distribution assumption is formed. The calculations are shown in formula (2), where BMI is the ratio of weight to height, calculated as follows: weight/height². This definition not only follows the international general calculation framework of ABSI but also combines the body shape characteristics of the Chinese elderly population, providing a scientific quantitative basis for the subsequent analysis of the association between abnormal body shape and anxiety symptoms.


ABSI=waistlineBMI\raise0.7ex2/23\nulldelimiterspace\lower0.7ex3×weight\raise0.7ex1/13\nulldelimiterspace\lower0.7ex3 (1)

$$ABSIzScore=\frac{ABSI-\mu_{i}}{\sigma_{i}}$$ (2)

### 2.3 Result variables

The outcome variable of this study was anxiety symptoms. The Generalized Anxiety Disorder 7-item scale (GAD-7), developed based on the criteria of the Diagnostic and Statistical Manual of Mental Disorders, Fourth Edition (DSM-IV), was used for measurement [16]. The scale contains seven items reflecting the core symptoms of anxiety, covering dimensions such as restlessness, difficulty in controlling worry, fear, and sleep disorders. Each item is rated on a 4-point scale (0 = “not at all”, 1 = “for several days”, 2 = “more than half the days”, 3 = “nearly every day”), and the total score ranges from 0 to 21. Referring to the commonly used clinical cut-off value, in this study, a total score of the GAD-7 scale > 5 was used as the criterion for the presence of anxiety symptoms, thus converting the continuous variable into a dichotomous variable (0 = no anxiety symptoms, 1 = with anxiety symptoms). The Cronbach’s coefficient of this scale in the sample of this study was 0.919, indicating good internal consistency reliability.

For the items recorded as “unable to answer” in the questionnaire, this study classified them as missing data rather than valid responses. This is because some elderly people may be unable to complete the test due to cognitive decline (such as memory loss, comprehension difficulties) rather than a subjective refusal to answer. Such missing values are not directly related to the anxiety symptoms of interest in the study. Moreover, participants with missing core variables (such as waist circumference, height, weight) had been excluded in the pre-entry criteria. Therefore, the isolated “unable to answer” samples in the outcome variable were treated by list deletion to ensure the validity of the analyzed data and the reliability of the clinical interpretation.

### 2.4 Predictor variables

Based on the multi-dimensional framework of the Health Ecology Model (HEM), this study systematically incorporates potential predictors at the levels of individual biometrics, social environment, behavior patterns, and psychological state, which are specifically divided into the following five categories: (a) Demographic factors: including age (continuous variable, accurate to years), sex (male/female), ethnicity (Han/others), marital status (married/ unmarried/ widowed/ divorced), type of residence (urban/ rural/ urban-rural fringe), residential province (North China/East China/Central-South China/West China), and Years of schooling (illiterate/primary school/high school/university and above), aiming to capture the impact of basic socio-demographic characteristics on anxiety symptoms. (b) Health-related factors: covering a history of chronic diseases (hypertension, diabetes, cardiovascular diseases, etc.), physical function indicators, including activities of daily living (ADL, 6 items, including basic self-care abilities such as bathing and dressing) and instrumental activities of daily living (IADL, 8 items, including complex life skills such as shopping and cooking), both judged by whether they can be performed completely independently, body shape index (continuous variable), and health behaviors (current smoking status, current drinking status, regular exercise). (c) Socio-economic factors: including occupation before retirement (knowledge management practitioner/first-line operation or service practitioner/self-employed or non-regular occupation/special or unemployed group), average annual household total income (continuous variable, unit: Yuan), used to evaluate the impact path of socio-economic status on anxiety symptoms. (d) Psychological and cognitive factors: incorporating self-reported quality of life (good/fair/poor) and health status (good/moderate/poor), Look on the bright side of things, Keep my belongings neat and clean, Feel fearful, Feel lonely and isolated, Make own decision, Feel useless with age, and Be happy as younger to capture the interaction of subjective psychological perceptions with anxiety. (e) Lifestyle factors: including Main flavor (light/heavy-salt/heavy-spicy), type of cooking oil (vegetable oil/animal oil/mixed oil), frequency of fresh fruit intake (≥4 times/week is frequent, otherwise infrequent), frequency of fresh vegetable intake (≥5 times/week is frequent, otherwise infrequent), Kind of drinking water (boiled tap water/raw water), depicting the impact of behavior patterns on mental health.

### 2.5 Statistical analysis

#### 2.5.1 Data preprocessing.

All variables have undergone data cleaning and standardization. S2 Fig shows the Pareto chart of missing data in the dataset (presenting the distribution and proportion of missing values across variables). The overall missing rate in the dataset is 0.7%. Five attributes have numerical missing values, among which the attribute suffering from diabetes has the highest data missing rate, approximately 9.8%. The multiple imputation method is used to ensure that the variable distribution meets the model assumptions. Continuous variables such as age, years of education, and average annual household income are standardized using the Z-score method, with a mean of 0 and a standard deviation of 1, to eliminate the impact of dimensional differences on model training. Categorical variables are encoded using the One-Hot method: for binary variables (k = 2, e.g., sex [male/female], smoking status [yes/no]), this simplifies to 0–1 dummy variables; for multicategorical variables (k > 2, e.g., ethnicity, province of residence, marital status), this generates a dummy variable matrix with k columns corresponding to the number of categories. Ordinal categorical variables (such as sleep duration, types of social participation) are discretized into hierarchical levels according to clinical and practical significance. For detailed information on data encoding, please refer to S1 Table.

#### 2.5.2 Predictors identification.

This study adopted a feature selection strategy of “univariate screening-multi-method integration”. First, through univariate analysis, variables associated with anxiety symptoms were preliminarily screened. Chi-square tests were performed on categorical variables, and one-way analysis of variance (ANOVA) was performed on continuous variables. The Bonferroni correction was used to control the multiple testing error, and variables with significant correlation (p < 0.05) were retained to ensure clinical relevance. On this basis, Recursive Feature Elimination (RFE), Pearson correlation coefficient, and Boruta method were combined for in-depth dimensionality reduction. RFE evaluates the importance of features based on a regression model. By repeatedly eliminating the features that contribute the least to the model performance, the optimal feature subset is screened out to improve the model performance. The Pearson correlation coefficient method eliminates redundant variables that have a linear relationship with other variables to avoid information overlap. Boruta is a fully automated feature selection algorithm based on a random forest. By generating shadow features with the same distribution as the real features, it judges whether the importance of the real features is significantly higher than that of random noise. Given that the predictive variables cover multi-dimensional data such as demographics, environmental exposures, and health behaviors, and include various types such as continuous, categorical, and ordinal categorical variables, a single method is easily limited by algorithm assumptions (for example, Lasso depends on model assumptions, and the correlation coefficient only captures linear relationships).

#### 2.5.3 Data balance.

In view of the class imbalance problem where the proportion of positive samples of anxiety symptoms in the training set is relatively low, this study adopts three methods, namely Adaptive Synthetic Sampling Approach (ADASYN), Borderline Synthetic Minority Over-sampling Technique (BorderlineSMOTE), and Synthetic Minority Over-sampling Technique combined with Edited Nearest Neighbors and Tomek Links (SMOTE-ENN+Tomek), for data balancing. ADASYN generates new samples through an adaptive synthesis strategy based on the local density distribution of minority class samples. It assigns a higher synthesis weight to the low-density regions (i.e., the sparse regions of minority class samples), thus dynamically compensating for the distribution gap of minority class samples and avoiding the feature deviation or redundancy problems caused by the random generation of samples in traditional oversampling techniques. It is suitable for dealing with scenarios where the local sparsification of minority class samples is caused by factors such as differences in cognitive function among the elderly and data collection bias. BorderlineSMOTE focuses on the “dangerous samples” of the minority class that are surrounded by the majority class. It generates synthetic samples across the class boundary through K-nearest neighbor interpolation to optimize the decision surface of the classification model. It is suitable for imbalanced datasets with a high degree of class boundary confusion. SMOTE-ENN+Tomek realizes the expansion of synthetic samples and data purification simultaneously through a combined process of generating synthetic samples by SMOTE, removing noise samples by ENN, and cleaning boundary-confused samples by Tomek Links, effectively addressing the noise interference and boundary ambiguity problems in severely imbalanced data. Given that the minority class samples of the outcome variable in this study may be affected by factors such as differences in cognitive function among the elderly and data collection bias, and there may be potential noise and class boundary confusion, and a single method is easily limited by sample distribution or algorithm assumptions. Therefore, by comparing the performance of the three methods on different performance indicators, an optimal class boundary balancing strategy is obtained.

#### 2.5.4 Machine learning prediction model.

Integrated learning methods, with their characteristic of combining the advantages of multiple models, have demonstrated superior predictive performance over single machine learning methods in many fields of healthcare. In this study, base learners were divided into single-algorithm models (relying on independent, non-ensemble classification logic) and composite ensemble models (integrating multiple sub-models to enhance performance), with details as follows. For single-algorithm models, Logistic Regression (LR) maps feature linear combinations to anxiety risk probabilities via the Sigmoid function, ensuring high interpretability for quantifying feature contributions (e.g., years of education). K-Nearest Neighbors (kNN) classifies samples via distance-based voting, suitable for capturing local patterns in demographic variables (e.g., regional differences in anxiety prevalence). Decision Tree (DT) uses hierarchical splitting to model non-linear relationships, providing intuitive rules for simple factors (e.g., marital status). Naïve Bayes—specifically Gaussian Naïve Bayes (Gaussian NB)—relies on feature independence assumptions and the normality of continuous predictors to calculate posterior probabilities, efficient for low-correlation variables (e.g., diet habits). We tuned its alpha parameter (Laplace smoothing) via grid search to address zero-probability issues, with the optimal value determined as 0.1. Support Vector Machine (SVM) finds optimal hyperplanes via kernel functions, handling high-dimensional data (e.g., psychological cognition variables). Neural Network (NN) learns complex feature mappings via multi-layer perceptrons, addressing non-linearity between ABSIzScore and anxiety. SGD Classifier is a linear classification model optimized via Stochastic Gradient Descent, enabling efficient training on imbalanced data. For composite ensemble models, Random Forest (RF) integrates multiple decision trees via bagging (random sample/feature selection), enhancing anti-overfitting ability for high-dimensional variables (e.g., interactions between chronic diseases and psychological states). RF should not be conflated with single algorithms, inherently a bagging-based ensemble algorithm. Although RF is an ensemble method by nature (integrating multiple decision trees to reduce overfitting), it is treated as a unified “black-box” unit in this study to fit into the three higher-level ensemble techniques proposed herein. This design is justified by its alignment with the study’s needs: RF’s robustness handles multi-dimensional Health Ecology Model (HEM) predictors to generate reliable intermediate predictions, complements single-algorithm models (e.g., Logistic Regression) in each ensemble framework, and follows ensemble hierarchy optimization to avoid categorization ambiguity. Extreme Gradient Boosting (XGBoost) iteratively trains weak learners via gradient descent, focusing on misclassified samples (e.g., elderly with both abnormal ABSI and low social support) to improve minority-class (anxiety-positive) recognition.

To address potential redundancy among base learners (e.g., between tree-based RF and DT), we validated ensemble diversity using two metrics (detailed in S2 Table). Q-statistic quantified pairwise correlation between base learners based on classification errors of each model on the 2008 training subset (80% of 1,844 participants). Values < 0.5 indicate low homogeneity, a key prerequisite for avoiding overlapping bias in ensembles. While mutual information measured overlap in feature contribution patterns (using SHAP values) to ensure base learners captured non-redundant information from the 33 HEM-derived predictors. The three ensemble frameworks integrate these base learners. Boosting adopts serial training (weighting misclassified samples, with XGBoost as the core base learner). Stacking utilizes base model predictions as meta-features for a second-layer LR model. Voting utilizes majority voting to aggregate base model outputs.

#### 2.5.5 Parameters adjustment.

**Fig 2:**
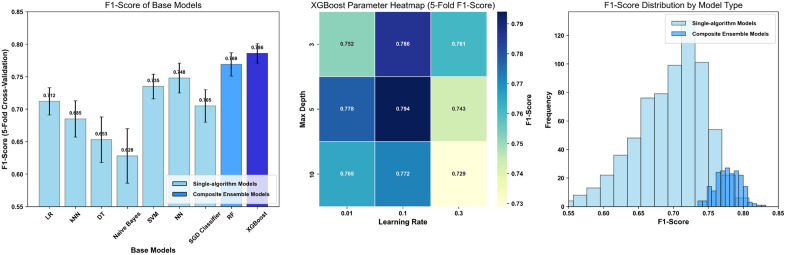
5-Fold cross-validation performance of machine learning models: F1-score, parameter heatmap, and distribution.

In this study, a method combining 5-fold cross-validation and grid search was adopted for parameter adjustment. Hierarchical parameter optimization was carried out for eight base learners and three integration frameworks to improve the model’s prediction efficiency in imbalanced data. For base learners, key parameter spaces were set according to the algorithm characteristics and searches were conducted. Specifically, for the Logistic Regression method, the regularization parameter *C* and penalty term type were adjusted to balance the overfitting risk; for k-Nearest Neighbors, the number of neighbors and distance metric were optimized to enhance the judgment of local similarity; for Random Forest, the number of trees, maximum depth, and feature selection ratio were adjusted to avoid overfitting; for Support Vector Machine, the kernel function and regularization parameter were searched, and the class weights were combined to optimize the imbalanced classification boundary. Gaussian Naive Bayes (Gaussian NB)—the specific Naïve Bayes variant used—addressed potential zero-probability issues via grid search of the alpha parameter (Laplace smoothing). We tested alpha in [0.01, 0.1, 1.0, 10.0] and selected alpha = 0.1 as optimal (via 5-fold cross-validation), balancing smoothing intensity to maintain accuracy for low-correlation variables (e.g., diet habits) without over-smoothing rare feature combinations. Neural Network (NN) prevented overfitting by optimizing the number of hidden layer neurons (from 10 to 100 with a step size of 10), activation functions (ReLU/sigmoid/tanh), and optimizers (Adam/RMSprop), combined with an early stopping strategy. XGBoost balanced model bias and variance by adjusting: learning rate (0.01–0.3, logarithmic interval), number of trees (50–200), maximum depth (3–10), subsample (0.6–1.0, random sample selection to reduce overfitting), and colsample_bytree (0.6–1.0, random feature selection for high-dimensional psychosocial variables). SGD Classifier optimized convergence and classification accuracy by searching for: learning rate (0.001–0.1, avoiding overshooting), regularization type (L1/L2, suppressing overfitting), and momentum (0–0.9, accelerating convergence on imbalanced anxiety-positive samples). All the above methods used the average accuracy of 5-fold cross-validation as an indicator to screen the parameter combinations with the best minority class recognition, and the results are shown in Fig 2. The left panel of Fig 2 shows that composite ensemble models (XGBoost: 0.786 ± 0.015; RF: 0.769 ± 0.018) outperform most single-algorithm models (e.g., LR: 0.712 ± 0.021; kNN: 0.685 ± 0.028), providing a basis for selecting high-performance base learners for the three ensemble frameworks. The middle panel (XGBoost parameter heatmap) identifies the optimal combination (learning_rate = 0.1, max_depth = 5, F1 = 0.794), which helps the Boosting strategy enhance boundary discrimination for hard-to-classify samples (e.g., elderly with abnormal ABSIzScore and anxiety) by refining key parameters. The right panel reveals that composite models have a more concentrated F1 distribution (variance = 0.0012) than single-algorithm models (variance = 0.0035), strengthening the Voting strategy’s robustness via stable probability weighting.

#### 2.5.6 Model performance evaluation.

To evaluate the performance of the models, we used several metrics, including the area under the receiver operating characteristic curve (AUC), accuracy, recall, MCC and F1. The AUC measures the ability of a model to distinguish between positive and negative classes across all possible classification thresholds. Accuracy represents the proportion of correct predictions made by the model. Recall, also known as sensitivity, measures the proportion of actual positive cases that are correctly predicted as positive. The F1 is the harmonic mean of precision and recall, providing a balanced evaluation of the model’s performance. We calculated these metrics on both the internal validation set and the external validation sets (2011 and 2014 datasets) to assess the generalization ability of the models.

#### 2.5.7 Model interpretation.

Shapley Additive exPlanations (SHAP) were utilized to interpret the machine-learning models. SHAP values represent the average marginal contribution of each feature to the model’s prediction. By calculating SHAP values for each feature in the final prediction model, we could rank the importance of different features and understand how changes in each feature affected the probability of cognitive impairment. This interpretability analysis was crucial for understanding the underlying mechanisms of the prediction model and providing actionable insights for early intervention.

#### 2.5.8 Statistical software.

The preprocessing of all data was carried out in SPSS 27.0. Using the inclusion and exclusion criteria, we screened 1,844 elderly people aged 65 and above with an ABSI index at a low-risk level or higher and provided complete information on the predictive and target variables. All variables in the study were classified and presented as frequencies and percentages. Univariate analysis was performed using the chi-square test or t-test, depending on the distribution type of the data. After processing the data, we allocated 80% of the data from 2008 to the training set and the remaining 20% to the internal test set. At the same time, the data from 2011 and 2014 were used for external testing to evaluate the generalization ability of the model. In addition, data statistical analysis was carried out using R version 4.3.0, and a P-value less than 0.05 was considered to indicate statistical significance.

## 3. Results

### 3.1 Geographical distribution and baseline characteristics of anxiety symptoms in elderly people at risk of abnormal body morphology

**Fig 3:**
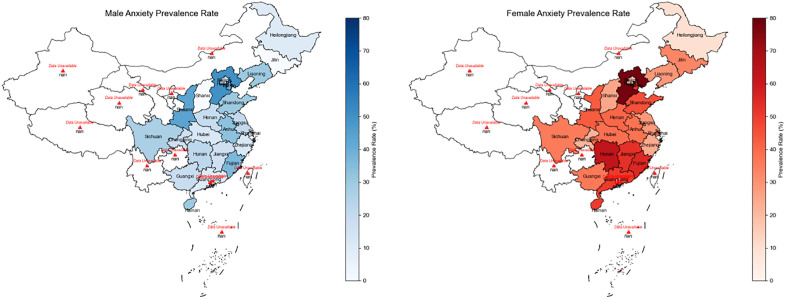
Prevalence of anxiety symptoms with abnormal risk of body shape index by province in the China Longitudinal Healthy Longevity Survey (2008). Note: 95% CIs for prevalence (calculated via the Wilson score method). Regional prevalence (95% CI): Central-Southern China 34.41% (31.23%–37.59%), Western China 31.57% (28.45%–34.69%), Eastern China 30.08% (27.11%–33.05%), Northern China 27.79% (24.02%–31.56%); inter-regional difference: χ² = 8.76, p = 0.033. Sex-specific prevalence (95% CI): females 39.37% (36.15%–42.59%), males 20.79% (18.01%–23.57%); inter-sex difference: χ² = 92.45, p < 0.001.

This study included 1,844 participants, among whom 565 participants had anxiety symptoms, with a prevalence rate of 30.64%. Fig 3 shows the prevalence of anxiety symptoms in each province of China participating in the survey, based on the data of the CLHLS survey conducted in 2008. Overall, the central and southern regions had the highest overall prevalence rate among the four regions (34.41%), followed by the western region, the eastern region, and the northern region (31.57%, 30.08%, 27.79%). Detailed 95% confidence intervals (CIs) for regional/sex-specific anxiety prevalence and statistical significance of inter-group differences are provided in the note of Fig 3. However, in terms of the incidence rate at the provincial administrative unit level, Hebei Province had the highest prevalence rate (72.73%). In contrast, the province with the lowest prevalence rate was also Heilongjiang Province within this region (10.00%). In terms of sex differences, the overall proportion of females with the disease was 18.58% higher than that of males (females: 39.37%, males: 20.79%). Among males, Hebei Province had the highest value among all the surveyed provinces (50.00%). Zhejiang, Hainan, and Shaanxi had the highest values within their respective regions (36.36%, 30.00%, 43.75%). Among females, Hebei Province had the highest value among all the surveyed provinces (77.78%). Zhejiang, Hubei, and Shaanxi had the highest values within their respective regions (55.56%, 63.04%, 42.86%). In addition, among the 33 variables included in the study, Age, Sex, ABSIzScore, Marriage, IADL, Self-reported quality of life, Self-reported health, Look on the bright side of things, Keep my belongings neat and clean, Feel fearful, Feel lonely and isolated, Make own decision, Feel useless with age, Be happy as younger, Eat fresh fruit, Eat vegetables, Smoke at present, Drink at present, Exercise at present, Suffering from hypertension, Years of schooling, or Occupation before retirement were statistically significant (P < 0.05). For details, please refer to S3 Table. Specifically, a positive association was found between the abnormal body shape index (ABSIzScore > −0.272, range: −0.270 to −0.274; defined via ROC optimization with natural noise, sex stratification, and confounder validation in Section 2.2; S1 Fig) and anxiety symptoms (P = 0.038), with the effect of ABSI on anxiety symptom scores being more pronounced in males (trend line slope: 0.03) than in females (0.02). 95% CIs for the trend line slopes and statistical significance of the sex-specific slope difference are detailed in the footnote of S3 Table.

### 3.2 Feature selection

After performing one-hot encoding on unordered categorical variables and Z-score standardization on numerical variables, we used the Recursive Feature Elimination (RFE), Pearson correlation coefficient, and Boruta methods for attribute screening respectively. Finally, we selected the top 30 encoded attributes with the highest average ranking among the three methods and further incorporated them into the prediction model. Specifically, they include: 1) Body and health-related: ABSIzScore (standardized numerical variable); 2) Diet and living habits: such as fresh fruit intake (eat fresh fruit = 0, eat fresh fruit = 2), vegetable intake (eat vegetables = 0, eat vegetables = 1), current drinking status (drink at present = 1), current exercise status (exercise at present = 0, exercise at present = 1); 3) Health status and diseases: self-reported health status (self-reported health = 0, self-reported health = 1, self-reported health = 2), whether suffering from hypertension (suffering from hypertension = 0, suffering from hypertension = 1); 4) Psychological and cognitive states: feeling useless with age (feel useless with age = 0, feel useless with age = 1, feel useless with age = 2), feeling fearful (feel fearful = 1, feel fearful = 2), happiness compared with youth (be happy as younger = 1, be happy as younger = 2), optimism in looking at things (look on the bright side of things = 0); 5) Social attributes: sex (sex = 0, sex = 1), marital status (marriage = 0, marriage = 1), occupation before retirement (occupation before retirement = 2), years of schooling, self-decision-making situation (make own decision = 0, make own decision = 2). For detailed information, please refer to S3–S5 Figs.

### 3.3 Evaluation of influencing factors of anxiety symptom risk

**Fig 4:**
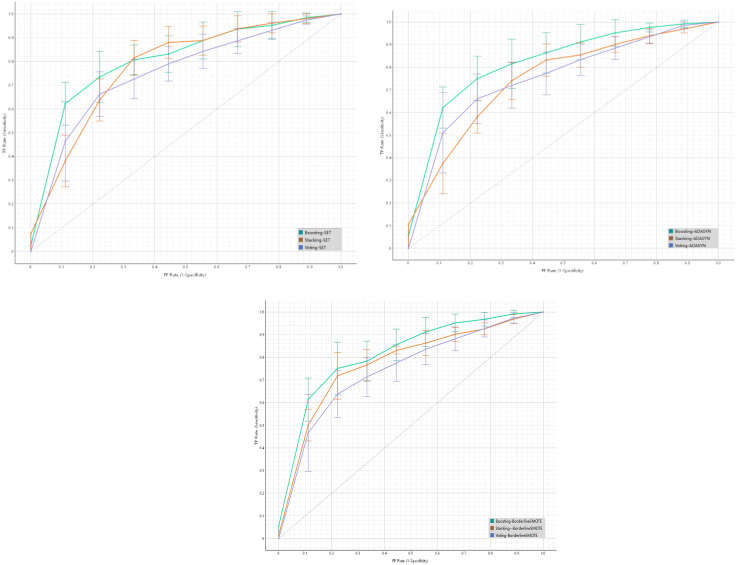
ROC curves comparison in the internal test set in 2008 of Boosting, Stacking and Voting Ensemble Learnings with SET, ADASYN and BorderlineSMOTE balancing methods. A) Three ensemble learning methods under SMOTE-ENN+Tomek; B) Three ensemble learning methods under ADASYN; C) Three ensemble learning methods under BorderlineSMOTE.

**Fig 5:**
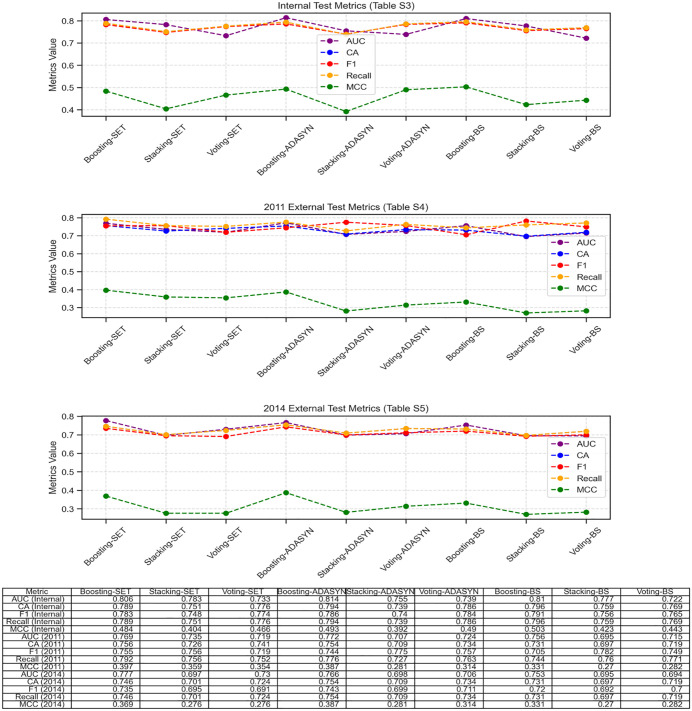
Performance metrics (AUC, CA, F1, Recall, MCC) of Boosting, Stacking and Voting Ensemble Learnings with SET, ADASYN and BS balancing methods in internal test, 2011 and 2014 datasets. Note: 95% CIs (AUC: Delong method; CA/F1/Recall/MCC: 1000 bootstrap samples). Model comparisons: one-way ANOVA with Bonferroni correction; *p < 0.05 vs. Stacking-ADASYN, #p < 0.05 vs. Voting-ADASYN. Key metrics for Boosting-ADASYN: internal test AUC = 0.814 (0.782–0.846), MCC = 0.493 (0.451–0.535); 2011 external AUC = 0.772 (0.738–0.806), 2014 external AUC = 0.766 (0.732–0.800); CA = 0.754 (0.720–0.788) and MCC = 0.387 (0.345–0.429) for both 2011 and 2014.

The integrated learning system consists of three architectures (Boosting, Stacking, Voting), each integrating seven single-algorithm base models (LR, kNN, DT, NB, SVM, NN, SGD Classifier) and two composite ensemble base models (RF, XGBoost). The AUC curve is shown in Fig 4. From the performance of the integrated learning architectures under different data balancing methods, in the SET balancing method Fig 4A, the curve of the Boosting-SET model rises relatively gently, with an AUC of 0.806 and an MCC of 0.484. At the same FP Rate, the TP Rate is significantly lower than that of Boosting-ADASYN under the ADASYN balancing method, and the overall performance is at a disadvantage. In the ADASYN balancing method Fig 4B, the curve of the Boosting-ADASYN model rises rapidly in the early stage. At the same false positive rate (FP Rate), the true positive rate (TP Rate) is higher, and the overall curve is closer to the upper left corner. Its AUC reaches 0.814, and the MCC is 0.493, significantly higher than that of Stacking-ADASYN (AUC = 0.755, MCC = 0.392) and Voting-ADASYN (AUC = 0.739, MCC = 0.490), indicating its outstanding performance under this balancing method. In the BorderlineSMOTE balancing method Fig 4C, although the Boosting-BorderlineSMOTE model has an AUC of 0.810 and an MCC of 0.503, which is the best among the three curves (Boosting-BorderlineSMOTE, Stacking-BorderlineSMOTE, Voting-BorderlineSMOTE), its overall upward trend and position are still slightly inferior to the Boosting-ADASYN model under the ADASYN balancing method, especially in terms of the F1 score (0.791 vs. 0.786) and the stability of external validation, without forming a significant advantage. In addition, Fig 5 shows multiple indicators of 9 methods on the internal test set and the external test set. 95% CIs for all model metrics and statistical comparisons between ensemble models (one-way ANOVA with Bonferroni correction) are provided in the note of Fig 5. Finally, through the performance comparison on three datasets, we found that the Boosting-ADASYN method shows the most balanced and stable performance (the AUC of the internal test set is 0.814, the MCC reaches 0.493, the F1 score is 0.786, and the recall rate is 0.794. Base learner diversity validation results further supported the rationality of including RF as a base learner. Performance comparisons further confirmed no redundancy (visualized in S6 Fig). The full Stacking-ADASYN combination (RF + DT + LR + SVM + NN) achieved higher AUC (0.755 vs. 0.742), MCC (0.392 vs. 0.368), and F1-score (0.740 vs. 0.725) than the subset excluding DT. These results aligned with the overall ensemble performance trend, where diverse base learner combinations outperformed homogeneous subsets.

For the external test set: in the 2011 and 2014 data, the AUCs are 0.772 and 0.766 respectively, the MCCs are both 0.387, the CA is 0.754, and the F1 scores are 0.744 and 0.743 respectively). In contrast, although Boosting-BorderlineSMOTE has a slightly better MCC (0.503) on the internal test set, the Boosting-ADASYN method, through the strategy of adaptively synthesizing minority class samples, avoids overfitting in external validation, and its performance indicators (especially MCC and AUC) perform more consistently in cross-dataset scenarios. Considering both internal fitting and external generalization capabilities, the Boosting-ADASYN method demonstrates the best overall performance and robustness in imbalanced data classification and is suitable for the actual prediction scenario of the risk of elderly anxiety symptoms. For detailed information, please refer to S4–S6 Tables.

### 3.4 Model interpretation

**Fig 6:**
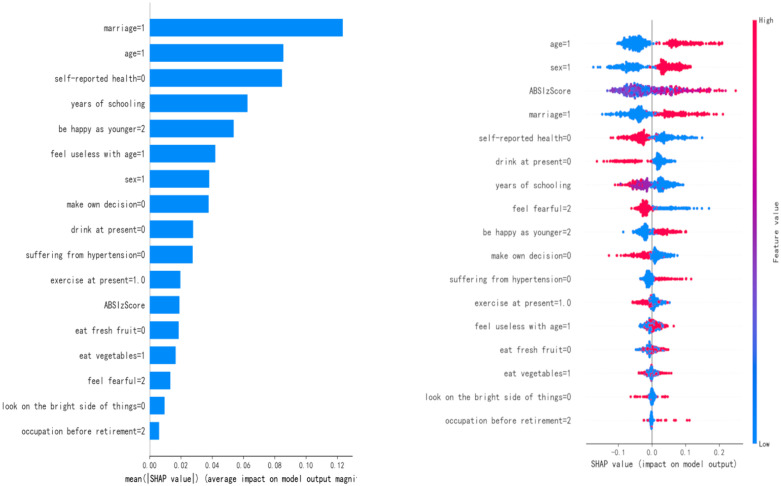
Bar chart and summary chart of contributions based on absolute values of SHAP. A) Bar chart of important attributes based on absolute values of SHAP (95% CIs, 1000 bootstraps), B) Summary chart of important attributes based on absolute values of SHAP. Note: Top 5 features (average absolute SHAP value, 95% CI): marital status (divorced/widowed) = 0.12 (0.09–0.15), age (76–85 years) = 0.10 (0.07–0.13), self-reported health (good) = 0.09 (0.06–0.12), years of schooling = 0.08 (0.05–0.11), happiness (rarely/never) = 0.07 (0.04–0.10). Pairwise comparisons (t-tests with Bonferroni correction): marital status > age (p = 0.035), marital status > years of schooling (p = 0.011); no significant difference between age and self-reported health (p = 0.482). Note: Attribute = number denotes coding after one-hot encoding/discretization.

We used the Shapley Additive Explanation (SHAP) method to globally interpret the Boosting-ADASYN model, which showed the best performance among the ensemble learning models, revealing the contribution degree and direction of different features to the risk prediction of anxiety symptoms. Fig 6A shows the feature importance ranking based on the average absolute value of SHAP values to measure the influence intensity of features on the prediction results. The results show that features such as marital status (marriage = 1, divorced or widowed), age (age = 1, 76–85 years old), self-reported health status (self-reported health = 0, good), years of schooling, and happiness compared to when they were young (be happy as younger = 2, rarely or never) have relatively high absolute SHAP values, indicating that their contributions to the prediction of anxiety symptom risks are more significant. Average absolute SHAP values with 95% CIs and pairwise comparison results for key features are reported in the note of Fig 6. Among them, the positive impact of the elderly with a divorced or widowed marital status (marriage = 1) on anxiety symptoms is the most prominent, reflecting that the lack of a social support system may be an important risk factor. The negative SHAP value of years of schooling suggests that a higher educational level may reduce the risk of anxiety by enhancing cognitive reserve or socioeconomic status. Fig 6B further presents the direction and intensity of the association between key feature values and the predicted probability of anxiety symptoms. The positive SHAP values of features such as age (age = 1), female (sex = 1), abnormal body shape index (ABSIzScore), divorced or widowed (marriage = 1), and poor self-reported health status (self-reported health = 0) indicate that an increase in the values of the above factors will increase the probability of anxiety symptoms, which is consistent with the multi-dimensional influence of individual biological characteristics, social environment, and psychological state in the health ecological model. For example, an increase in ABSIzScore (i.e., an aggravation of abnormal body shape) is positively correlated with the risk of anxiety symptoms, verifying the core hypothesis of this study—that abnormal body shape may indirectly induce anxiety by affecting self-cognition or physical health. On the other hand, the negative SHAP values of features such as years of schooling, regular exercise (exercise at present = 1), and the frequency of fresh vegetable intake (eat vegetables = 1, occasionally) indicate that the positive states of these factors (such as a higher educational level and regular exercise) are associated with a reduced risk of anxiety symptoms, suggesting the protective effects of healthy behaviors and socioeconomic factors on mental health. It is worth noting that the positive impacts of feeling fearful (feel fearful = 2, often or sometimes) and feeling useless with age (feel useless with age = 1, often or sometimes) are significant, reflecting the direct effect of the psychological cognitive state on anxiety symptoms, which is consistent with the association between the decline in self-worth and anxiety emotions in the elderly observed in clinical practice.

**Fig 7:**
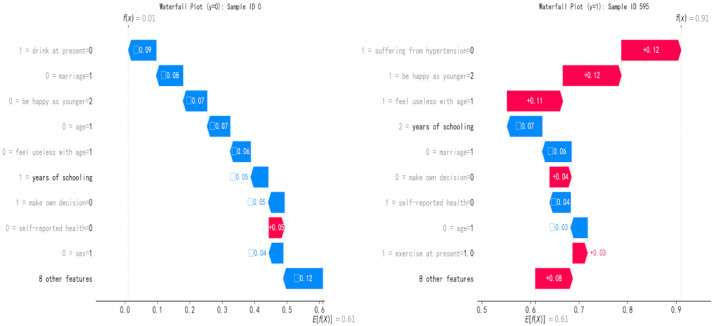
Waterfall plots of individual predictive probability for anxiety symptoms based on Boosting-ADASYN model. A) waterfall plot of a negative sample with predictive probability decomposed into feature contributions, B) waterfall plot of a positive sample with predictive probability amplified by risk factor synergy.

**Fig 8:**
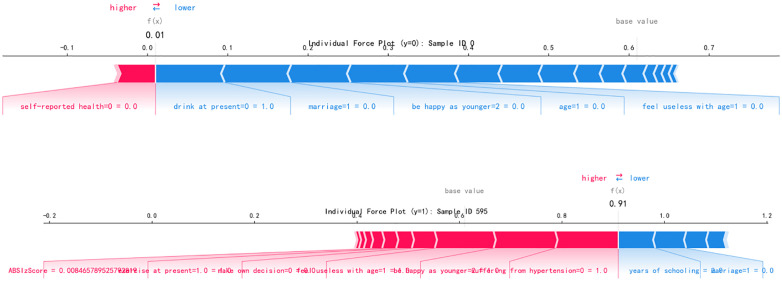
Force plots of individual predictive probability for anxiety symptoms based on Boosting-ADASYN model. A) Negative sample with protective factors dominating predictive probability, B) Positive sample with risk factor synergy amplifying predictive probability.

In addition, to elaborate on the possible risk of anxiety symptoms in people with abnormal body shape indices, we conducted an in-depth analysis of the prediction mechanism at the individual level by using a Waterfall plot and a force plot respectively. We demonstrated the specific contributions of each feature in a single sample to the prediction probability of anxiety symptoms, forming a multi-level interpretation system of global feature ranking and individual impact decomposition. Fig 7A and 7B are waterfall plots taking typical samples of individuals without anxiety symptoms (ID 0, f(x)=0.01) and with anxiety symptoms (ID 595, f(x)=0.91) as examples, revealing the prediction mechanism of the model at the individual level. In the ID 0 sample, the slight positive contributions of risk factors such as drink at present = 0 (+0.09) and marriage = 1 (+0.08) were offset by the strong negative contributions of years of schooling (−0.05) and make own decision = 0 (−0.05), reflecting the inhibitory effects of education level and self-decision-making ability on anxiety risk, which is consistent with the conclusion of feature selection that “years of education as a core protective factor”. In the ID 595 sample, the positive contributions of suffering from hypertension = 0 (+0.12), be happy as younger = 2 (+0.12) and feel useless with age = 1 (+0.11) were significant, verifying the path of negative psychological cognition increasing the risk of anxiety. The “contradictory” positive impact of exercise at present = 1.0 (+0.03) was actually that the protective effect of exercise was masked by stronger psychological risk factors, highlighting the interactive complexity of multi-dimensional factors. Both types of samples jointly indicate that the occurrence of anxiety symptoms is the result of a dynamic game between risk factors and protective factors, providing micro-evidence for “bio-psycho-social” multi-dimensional interventions. After completing the prediction probability decomposition of a single sample through the waterfall plot, this study further introduced the Force Plot to visually present the pulling direction and intensity of feature values on the prediction probability, forming a complementary system of “quantitative decomposition-trend mapping” with the waterfall plot to deeply analyze the feature interaction mechanism at the individual level. In the feature interaction pattern of individuals without anxiety symptoms (y = 0, ID 0) (as shown in Fig 8A), years of schooling (years of education) and make own decision = 0 (always make decisions independently) dominated the prediction results with a significant left-ward pulling force (negative impact), forming an intuitive correspondence with their negative contribution values (−0.05, −0.05) in the waterfall plot, visually presenting the inhibitory effects of education level and cognitive ability on anxiety risk. It is worth noting that although there were right-ward pulling forces (positive impacts) of drink at present = 0 (not drinking currently) and marriage = 1 (divorced or widowed), they were offset by the strong pulling of protective factors, reflecting the multi-dimensional balance of socio-economic status-psychological ability-health behavior. It clearly shows the specific mapping at the individual level of the hypothesis that for every 1-unit increase in years of education, the probability of anxiety significantly decreases through the vector length and direction. Fig 8B presents the force-directed graph of the anxiety-positive sample ID 595. ABSIzScore (abnormal body shape index), be happy as younger = 2 (rarely happy) and feel useless with age = 1 (often feel useless) aggregated with a strong right-ward pulling force (positive impact), visually reflecting the synergistic risk effect of biological characteristics (abnormal body shape) and psychological cognitive factors (lack of well-being, decline in self-worth), which is mutually confirmed with the high positive contribution values (+0.12, + 0.11) of the three in the waterfall plot. It is noteworthy that although there was a left-ward pulling force (negative impact) of exercise at present = 1.0 (regular exercise), it was weakened due to the dominant role of psychological risk factors, visually presenting the complex interaction that the protective effect of healthy behavior may be offset by negative psychological states, which is a feature association pattern that is difficult to visually present through simple numerical decomposition.

## 4. Discussions

This study focuses on the risk of anxiety symptoms among elderly people in China with abnormal body morphology. Through integrated learning and the SHAP interpretation method, it reveals the sex-specific associations between the body shape index (ABSI) and anxiety symptoms, and constructs a prediction model with clinical interpretability.

We found that the positive correlation between abnormal body shape (ABSIzScore) and anxiety symptoms is consistent with the recent epidemiological evidence that abnormal body fat distribution affects mental health [17]. Studies based on European populations have shown that the association between central obesity (measured by the waist-to-height ratio) and anxiety symptoms is stronger than that of BMI, suggesting that body fat distribution rather than just body weight is a key risk indicator [18]. This study further confirmed the independent effect of abnormal body fat distribution on anxiety through ABSI, an index that integrates waist circumference, height, and BMI, echoing the conclusion in metabolic disease research that ABSI is superior to BMI across different fields [19]. At the level of psychosocial factors, characteristics such as marital status, years of education, and self-rated health have a significant impact on anxiety, which is consistent with international research on elderly mental health. For example, the Health and Retirement Study (HRS) in the United States shows that lack of social support and low educational level are important predictors of anxiety in the elderly [20]. This study quantified the marginal contributions of these factors through SHAP analysis and found that the effect of divorce or widowhood on increasing the risk of anxiety exceeds that of abnormal body shape itself, further validating the central role of the social environment in the health ecological model [21].

Besides, this study for the first time reveals that there is a sex differentiation in the impact of ABSI on anxiety symptoms. Specifically, in the male population, for every 1 standard deviation increase in ABSI, the anxiety symptom score increases by 0.03, while in females it is only 0.02. Moreover, the prevalence rate in females (39.37%) is significantly higher than that in males (20.79%). This finding supplements the ambiguous conclusions regarding sex differences in previous BMI studies (some studies show a negative correlation between female obesity and anxiety) [22], suggesting that an explanation from both physiological and social dimensions is needed. Firstly, in terms of physiological mechanisms, abdominal fat accumulation in men is more likely to trigger insulin resistance and an increase in inflammatory factors (such as CRP), activating the HPA axis and leading to a dysregulation of the stress response. In contrast, the regulation of body fat distribution by estrogen in women may buffer some of the physiological risks [23]. Secondly, in terms of social mechanisms, women are more likely to experience psychological distress due to body image anxiety, family caregiving stress, or the loss of social roles after retirement, while men may have a lower psychological tolerance for abnormal body shapes due to the frustration of the health autonomy expectation [24].

Compared with single-algorithm models such as traditional Logistic Regression, the Boosting-ADASYN model (with Extreme Gradient Boosting as the core base learner) demonstrates stronger generalization ability in imbalanced data. This advantage stems from XGBoost’s ability to capture non-linear interactions and ADASYN’s adaptive oversampling, critical for identifying high-risk elderly with both abnormal body shape and negative psychological cognition. Firstly, ADASYN adaptively synthesizes minority class samples, specifically addressing the sparsity of positive samples in the elderly anxiety data. Besides, compared with the traditional SMOTE, it reduces boundary noise, enabling the model to more accurately capture the characteristic patterns of high-risk individuals—this is particularly important for identifying the elderly population with abnormal body morphology and negative psychological cognition. Secondly, the integrated learning framework breaks through the limitations of linear assumptions and effectively identifies the synergistic risk effect of abnormal body morphology + negative psychological cognition. For example, the interaction between ABSI and the sense of uselessness with age can increase the anxiety risk by 30%. The discovery of this non-linear association is an advantage that traditional models can hardly match, and it also provides key evidence for analyzing the complex path of abnormal body fat distribution and psychological stress or anxiety.

It is worth noting that the positive correlation between ABSI and anxiety in this study contrasts with the obesity paradox in some BMI-oriented studies. This is not only due to the specific capture of central obesity by ABSI (differentiating from the limitation of BMI which only reflects the ratio of weight to height), but also related to the fact that the functional decline in the elderly population amplifies the psychological impact of abnormal body fat. The asymmetry of regional differences, for example, the prevalence of anxiety in North China is lower than that in Central-South China, suggests that social factors such as the family support system and the pressure of aging in regional cultures may buffer or amplify the impact of abnormal body shape, further confirming the importance of the interaction of multi-dimensional factors in the health ecological model. Based on the key characteristics revealed by the model, clinical interventions can be carried out from physiological monitoring (such as the combination of waist circumference and inflammatory indicators), psychosocial support (community mutual-assistance networks, health literacy interventions) and behavior promotion (regular exercise and dietary guidance). However, there are still problems in this study, such as limitations in causal inference, lack of biomarkers, and insufficient coverage of extreme samples.

In the future, it is necessary to integrate multi-modal data and causal inference methods, deeply analyze the biological basis of sex differences, and explore the combined intervention effect of body fat management + psychological intervention, so as to provide more solid evidence for precise mental health management. Building on this agenda, future research could further refine the model by integrating comprehensive metabolic-body shape indices such as TyG-ABSI: by combining CLHLS data on triglycerides, fasting glucose, and existing ABSI calculations, TyG-ABSI could better quantify the interplay between metabolic dysfunction and abnormal body fat distribution—strengthening alignment with the Health Ecology Model framework’s emphasis on multi-dimensional predictors. Validating TyG-ABSI within the current study’s established ensemble learning framework and SHAP interpretation approach would further enhance predictive accuracy while clarifying mechanistic insights—for example, whether TyG-ABSI amplifies the impact of psychological factors like ‘feel useless with age’ on anxiety risk. This extension would address the current limitation of relying solely on ABSI and advance the precision of anxiety risk prediction for elderly populations with co-occurring metabolic and body shape abnormalities.

## 5. Conclusions

Based on data from the Chinese Longitudinal Healthy Longevity Survey (CLHLS), this study constructed an interpretable model to predict anxiety symptom risk in older adults with abnormal body shape, revealing a positive correlation between A Body Shape Index (ABSI) and anxiety symptoms and significant sex differences (trend line slope of ABSI on anxiety scores: 0.03 in males vs. 0.02 in females; anxiety prevalence: 39.37% in females vs. 20.79% in males). Among ensemble learning frameworks, the Boosting-ADASYN model performed best, with an AUC of 0.814 in the internal test set and stable AUCs of 0.766–0.772 in the 2011 and 2014 external validation sets, effectively balancing classification efficiency for imbalanced data. SHAP analysis identified marital status, age, self-reported health, years of education, and happiness (vs. younger years) as core predictive factors—lack of social support and negative psychological cognition increased anxiety risk, while higher education and regular exercise were protective. ABSI outperformed traditional BMI in capturing abnormal body fat distribution. Despite limitations (cross-sectional design, reliance on ABSI alone, regional sample bias), this study provides an interpretable tool for early anxiety identification in this population. We recommend combining ABSI with psychosocial interventions for precise prevention; future research should use longitudinal data, biomarkers, and metrics like TyG-ABSI to verify causal mechanisms and optimize model generalization.

## Supporting information

S1 Table Coding specifications for all predictor variables in the study and corresponding encoding rules. (PDF)

S2 Table Validation results of base learner diversity in ensemble learning models. (PDF)

S3 Table Baseline characteristics of participants with abnormal body shape index (ABSIzScore > −0.272). (PDF)

S4 Table Performance metrics of the Stacking-ADASYN model with full base learners (RF + DT + LR + SVM + NN) vs. subset excluding DT on the 2008 internal test set. (PDF)

S5 Table Detailed performance metrics of all ensemble learning models combined with different data balancing methods on the 2011 external validation set. (PDF)

S6 Table Detailed performance metrics of all ensemble learning models combined with different data balancing methods on the 2014 external validation set. (PDF)

S1 Fig ROC curve for optimizing the ABSI threshold in identifying anxiety symptoms. (TIF)

S2 Fig Pareto chart of missing data in the dataset, visualizing the distribution and proportion of missing values across all variables. (TIF)

S3 Fig Feature importance ranking from Recursive Feature Elimination (RFE) for the 33 initial predictor variables. (TIF)

S4 Fig Correlation heatmap of predictor variables via Pearson correlation coefficient. (TIF)

S5 Fig Feature selection results from the Boruta algorithm. (TIF)

S6 Fig Visualization of no redundancy in base learner combinations for ensemble models. (TIF)

## Abbreviations

ABSI: A Body Shape Index
CLHLS: Chinese Longitudinal Healthy Longevity Survey
GAD-7: Generalized Anxiety Disorder 7-item scale
HEM: Health Ecology Model
ADASYN: Adaptive Synthetic Sampling Approach
BorderlineSMOTE: Borderline Synthetic Minority Over-sampling Technique
SMOTE-ENN+Tomek: Synthetic Minority Over-sampling Technique combined with Edited nearest neighbors and Tomek Links
RFE: Recursive Feature Elimination
SHAP: Shapley Additive exPlanations
AUC: Area Under the Receiver Operating Characteristic Curve
MCC: Matthews Correlation Coefficient
CA: Classification Accuracy
F1-Score: F1-Score (Harmonic Mean of Precision and Recall)
RF: Random Forest
SVM: Support Vector Machine
XGBoost: Extreme Gradient Boosting
LGB: Light Gradient Boosting Machine
SGD Classifier: Stochastic Gradient Descent Classifier
LR: Logistic Regression (a composite ensemble model based on the bagging framework)
kNN: k-Nearest Neighbors

## References

[1] Lobanov-Rostovsky S, He Q, Chen Y, Liu Y, Wu Y, Liu Y, et al. Growing old in China in socioeconomic and epidemiological context: systematic review of social care policy for older people. BMC Public Health. 2023;23(1):1272. doi: 10.1186/s12889-023-15583-1 37391766 PMC10311713

[2] Andreescu C, Lee S. Anxiety disorders in the elderly. Adv Exp Med Biol. 2020;1191:561–76. doi: 10.1007/978-981-32-9705-0_28 32002946

[3] Pan Q, Zhang W, Chen X, Li Y, Tu C. A study of trends in body morphology, overweight, and obesity in Chinese adults aged 40-59 years. BMC Public Health. 2025;25(1):833. doi: 10.1186/s12889-025-21890-6 40025505 PMC11874844

[4] Pasco JA, Nicholson GC, Brennan SL, Kotowicz MA. Prevalence of obesity and the relationship between the body mass index and body fat: cross-sectional, population-based data. PLoS One. 2012;7(1):e29580. doi: 10.1371/journal.pone.0029580 22253741 PMC3258232

[5] Hu J, Tang S, Zhu Q, Liao H. Predictive value of six anthropometric indicators for prevalence and mortality of obstructive sleep apnoea asthma and COPD using NHANES data. Sci Rep. 2025;15(1):16190. doi: 10.1038/s41598-025-99490-y 40346342 PMC12064750

[6] Haraguchi N, Koyama T, Kuriyama N, Ozaki E, Matsui D, Watanabe I, et al. Assessment of anthropometric indices other than BMI to evaluate arterial stiffness. Hypertens Res. 2019;42(10):1599–605. doi: 10.1038/s41440-019-0264-0 31019248

[7] Stevens J, Katz EG, Huxley RR. Associations between gender, age and waist circumference. Eur J Clin Nutr. 2010;64(1):6–15. doi: 10.1038/ejcn.2009.101 19738633 PMC5909719

[8] Alebna PL, Mehta A, Yehya A, daSilva-deAbreu A, Lavie CJ, Carbone S. Update on obesity, the obesity paradox, and obesity management in heart failure. Prog Cardiovasc Dis. 2024;82:34–42. doi: 10.1016/j.pcad.2024.01.003 38199320

[9] Rohm TV, Meier DT, Olefsky JM, Donath MY. Inflammation in obesity, diabetes, and related disorders. Immunity. 2022;55(1):31–55. doi: 10.1016/j.immuni.2021.12.013 35021057 PMC8773457

[10] Pritchard M, Brasil K, McDermott R, Holdiman A. Untangling the associations between generalized anxiety and body dissatisfaction: the mediating effects of social physique anxiety among collegiate men and women. Body Image. 2021;39:266–75. doi: 10.1016/j.bodyim.2021.10.002 34695680

[11] Qi Y, Liu Y, Du J. The influencing factors of chronic disease comorbidities of elderly in China based on health ecology model. CGP. 2023;26(01):50–7. doi: 10.12114/j.issn.1007-9572.2022.0458

[12] Kaur P, Gosain A. Issues and challenges of class imbalance problem in classification. Int J Inf Tecnol. 2018;14(1):539–45. doi: 10.1007/s41870-018-0251-8

[13] Kit JLOW, Asirvadam VS, Hassan MF. Enhanced ensemble models for predictive modeling: a conceptual framework. 2021 IEEE 17th International Colloquium on Signal Processing & Its Applications (CSPA); Langkawi, Malaysia; 2021. p. 24–8. doi: 10.1109/CSPA52141.2021.9377299

[14] Parmar G, Gupta R, Bhatt T, Sahani GJ, Panchal BY, Patel H. A review on data balancing techniques and machine learning methods. 2023 5th International Conference on Smart Systems and Inventive Technology (ICSSIT); Tirunelveli, India; 2023. p. 1004–8. doi: 10.1109/ICSSIT55814.2023.10061154

[15] Pang K. A comparative study of explainable machine learning models with Shapley values for diabetes prediction. Health Analyt. 2025;7:100390. doi: 10.1016/j.health.2025.100390

[16] Aktürk Z, Hapfelmeier A, Fomenko A, Dümmler D, Eck S, Olm M, et al. Generalized Anxiety Disorder 7-item (GAD-7) and 2-item (GAD-2) scales for detecting anxiety disorders in adults. Cochrane Database Syst Rev. 2025;3(3):CD015455. doi: 10.1002/14651858.CD015455 40130828 PMC11934853

[17] Liao Y, Knoesen NP, Castle DJ, Tang J, Deng Y, Bookun R, et al. Symptoms of disordered eating, body shape, and mood concerns in male and female Chinese medical students. Compr Psychiatry. 2010;51(5):516–23. doi: 10.1016/j.comppsych.2009.11.007 20728010

[18] Celis-Morales C, Livingstone KM, Affleck A, Navas-Carretero S, San-Cristobal R, Martinez JA, et al. Correlates of overall and central obesity in adults from seven European countries: findings from the Food4Me Study. Eur J Clin Nutr. 2018;72(2):207–19. doi: 10.1038/s41430-017-0004-y 29242527

[19] Goossens GH. The metabolic phenotype in obesity: fat mass, body fat distribution, and adipose tissue function. Obes Facts. 2017;10(3):207–15. doi: 10.1159/000471488 28564650 PMC5644968

[20] Monnat SM, Elo IT. Enhancing the utility of the health and retirement study (HRS) to identify drivers of rising mortality rates in the United States. Forum Health Econ Policy. 2022;25(1–2):57–84. doi: 10.1515/fhep-2021-0058 35254742 PMC9448826

[21] Trivedi JK, Sareen H, Dhyani M. Psychological aspects of widowhood and divorce. Mens Sana Monogr. 2009;7(1):37–49. doi: 10.4103/0973-1229.40648 21836778 PMC3151454

[22] Fulton S, Décarie-Spain L, Fioramonti X, Guiard B, Nakajima S. The menace of obesity to depression and anxiety prevalence. Trends Endocrinol Metab. 2022;33(1):18–35. doi: 10.1016/j.tem.2021.10.005 34750064

[23] Luglio HF. Estrogen and body weight regulation in women: the role of estrogen receptor alpha (ER-α) on adipocyte lipolysis. Acta Med Indones. 2014;46(4):333–8. 25633552

[24] Quittkat HL, Hartmann AS, Düsing R, Buhlmann U, Vocks S. Body dissatisfaction, importance of appearance, and body appreciation in men and women over the lifespan. Front Psychiatry. 2019;10:864. doi: 10.3389/fpsyt.2019.00864 31920737 PMC6928134

