# Supplementary material for: Interpretable ensemble learning model with shapley additive explanations for predicting anxiety symptoms risk in Chinese older adults with body shape index abnormality
Source: PLoS One. 2025 Oct 30;20(10):e0335437. doi: 10.1371/journal.pone.0335437 (PMC12574866; doi:10.1371/journal.pone.0335437)
Supplement: S1 Table — (PDF) [file pone.0335437.s001.pdf]

Table S1
Assignments of independent variables.

Independent variables	Assignment
Age	0=66-75,1= 76-85,2=More than 85
Prov	0=North China { 11,12,13,14,21,22,23},1=East China = {31, 32, 33, 34, 35, 36, 37}, 2=Central and South ={41,42,43,44,45,46},3= West China ={50,51,61}
Residence	0= city 1= rural area 2= urban - rural fringe
Sex	0= male 1= female
Nation	0= Han ethnicity 1= ethnic minorities
Marriage	0= married 1= divorce or bereavement of a spouse 2= never been married
IADL	1=yes 0=no
ADL	1=yes 0=no
Self-reported quality of life	0= good 1=average 2=not good 3= not able to answer
Self-reported health	0= good 1=average 2=not good 3= not able to answer
Look on the bright side of things	0=always 1=often or sometimes 2=seldom or never 3= not able to answer
Keep my belongings neat and clean	0=always 1=often or sometimes 2=seldom or never 3= not able to answer
Feel fearful	1=always 2=often or sometimes 3=seldom or never 4= not able to answer
Feel lonely and isolated	1=always 2=often or sometimes 3=seldom or never 4= not able to answer
Make own decision	1=always 2=often or sometimes 3=seldom or never 4= not able to answer
Feel useless with age	1=always 2=often or sometimes 3=seldom or never 4= not able to answer
Be happy as younger	1=always 2=often or sometimes 3=seldom or never 4= not able to answer
Eat fresh fruit	1=quite often 2= occasionally 3=rarely or never
Eat vegetables	1=quite often 2= occasionally 3=rarely or never
Kind of grease for cooking	1=vegetable grease 2=gingili grease 3=lard 4=other animal's fat
Main flavor	1=insipidity 2=salty or sweet 3=hot or crude 4=do not have all the above tastes
Kind of drinking water	1=boiled water 2=un-boiled water
Smoke at present	1=yes 2=no
Drink at present	1=yes 2=no

Exercise at present	1=yes 2=no
Suffering from hypertension	1=yes 2=no 3= don't know
Suffering from diabetes	1=yes 2=no 3= don't know
Suffering from heart disease	1=yes 2=no 3= don't know
Suffering from stroke or cvd	1=yes 2=no 3= don't know
Suffering from arthritis	1=yes 2=no 3= don't know
Household income	1=between 0 and 10000 2=between 10001 and 50000 3=more than 50001
Years of schooling	0=illiterate 1=primary school stage 2= high school stage 3= university and above
Occupation before retirement	1=knowledge /management practitioner 2=front-line operation /service practitioner 3=autonomous/non-employed practitioners 4=special/non-employment groups
ABSIzScore	1=between -0.272 and 0.229 2=between 0.230 and 0.798 3=more than 0.798

Note: ADL, activities of daily living; IADL, instrumental activities of daily living; ABSIzScore, Z-Score for A Body Shape Index;
