# Supplementary material for: Interpretable ensemble learning model with shapley additive explanations for predicting anxiety symptoms risk in Chinese older adults with body shape index abnormality
Source: PLoS One. 2025 Oct 30;20(10):e0335437. doi: 10.1371/journal.pone.0335437 (PMC12574866; doi:10.1371/journal.pone.0335437)
Supplement: S2 Table — (PDF) [file pone.0335437.s002.pdf]

Table S2

Base Learner Diversity Metrics

Base Learner Pair	Q-Statistic	Mutual Information (Feature contribution overlap)
LR vs. kNN	0.15	0.12
LR vs. DT	0.18	0.14
LR vs. RF	0.23	0.16
DT vs. RF	0.21	0.18
RF vs. XGBoost	0.25	0.20
kNN vs. SVM	0.19	0.13
NN vs. SGD Classifier	0.22	0.15
