# Supplementary material for: Interpretable ensemble learning model with shapley additive explanations for predicting anxiety symptoms risk in Chinese older adults with body shape index abnormality
Source: PLoS One. 2025 Oct 30;20(10):e0335437. doi: 10.1371/journal.pone.0335437 (PMC12574866; doi:10.1371/journal.pone.0335437)
Supplement: S3 Table — (PDF) [file pone.0335437.s003.pdf]

Table S3

Selected characteristics of CLHLS participants.

Characteristic	n(%) non-anxiety symptom (n =1279)	n(%) anxiety symptom (n =565)	n(%) overall (N=1844)	*P* value
**Age**				<0.001
66-75	898 (83.23%)	181 (16.77%)	1079 (58.51%)	
76-85	327 (56.48%)	252 (43.52%)	579 (31.40%)	
More than 85	54 (29.03%)	132 (70.97%)	186 (10.09%)	
**Sex**				<0.001
Male	686 (79.21%)	180 (20.79%)	866 (46.96%)	
Female	593 (60.63%)	385 (39.37%)	978 (53.04%)	
**ABSIzScore (Numeric)**				0.038
Mean: -0.0250, Std: 0.7376, Min: -0.2719, Max: 1.5294				
**Province**				0.402
North China	111 (75.00%)	37 (25.00%)	148 (8.03%)	
East China	470 (68.51%)	216 (31.49%)	686 (37.20%)	
Central and Southern	514 (68.53%)	236 (31.47%)	750 (40.67%)	
West China	184 (70.77%)	76 (29.23%)	260 (14.10%)	
**Residence**				0.098
City	170 (74.56%)	58 (25.44%)	228 (12.36%)	
Rural area	258 (71.07%)	105 (28.93%)	363 (19.69%)	
Urban-rural fringe	851 (67.92%)	402 (32.08%)	1253 (67.95%)	
**Nation**				0.994
Han ethnicity	1197 (69.39%)	528 (30.61%)	1725 (93.55%)	
Ethnic minorities	82 (68.91%)	37 (31.09%)	119 (6.45%)	
**Marriage**				<0.001
Married	885 (78.39%)	244 (21.61%)	1129 (61.23%)	
Divorce or bereavement of a spouse	380 (54.52%)	317 (45.48%)	697 (37.80%)	
Never married	14 (77.78%)	4 (22.22%)	18 (0.98%)	

**ADL**				0.191
No	118 (64.84%)	64 (35.16%)	182 (9.87%)	
Yes	1161 (69.86%)	501 (30.14%)	1662 (90.13%)	
**IADL**				<0.001
No	5 (15.62%)	27 (84.38%)	32 (1.74%)	
Yes	1274 (70.31%)	538 (29.69%)	1812 (98.26%)	
**Self-reported quality of life**				<0.001
Very good	727 (70.38%)	306 (29.62%)	1033 (56.02%)	
General	478 (71.34%)	192 (28.66%)	670 (36.33%)	
Not good	65 (55.08%)	53 (44.92%)	118 (6.40%)	
Unable to answer	9 (39.13%)	14 (60.87%)	23 (1.25%)	
**Self-reported health**				<0.001
Very good	783 (76.92%)	235 (23.08%)	1018 (55.21%)	
General	368 (66.19%)	188 (33.81%)	556 (30.15%)	
Not good	120 (48.39%)	128 (51.61%)	248 (13.45%)	
Unable to answer	8 (36.36%)	14 (63.64%)	22 (1.19%)	
**Look on the bright side of things**				0.020
Always	182 (78.45%)	50 (21.55%)	232 (12.58%)	
Often or sometimes	1040 (69.47%)	457 (30.53%)	1497 (81.18%)	
Seldom or never	48 (53.93%)	41 (46.07%)	89 (4.83%)	
Unable to answer	9 (34.62%)	17 (65.38%)	26 (1.41%)	
**Keep my belongings neat and clean**				<0.001
Always	181 (72.69%)	68 (27.31%)	249 (13.50%)	
Often or sometimes	1073 (69.63%)	468 (30.37%)	1541 (83.57%)	
Seldom or never	17 (56.67%)	13 (43.33%)	30 (1.63%)	
Unable to answer	8 (33.33%)	16 (66.67%)	24 (1.30%)	
**Feel fearful**				<0.001
Always	22 (73.33%)	8 (26.67%)	30 (1.63%)	

Often or sometimes	251 (59.90%)	168 (40.10%)	419 (22.72%)	
Seldom or never	990 (72.69%)	372 (27.31%)	1362 (73.86%)	
Unable to answer	16 (48.48%)	17 (51.52%)	33 (1.79%)	
**Feel lonely and isolated**				<0.001
Always	11 (52.38%)	10 (47.62%)	21 (1.14%)	
Often or sometimes	242 (59.17%)	167 (40.83%)	409 (22.18%)	
Seldom or never	1012 (73.17%)	371 (26.83%)	1383 (75.00%)	
Unable to answer	14 (45.16%)	17 (54.84%)	31 (1.68%)	
**Make own decision**				<0.001
Always	553 (77.89%)	157 (22.11%)	710 (38.50%)	
Often or sometimes	615 (67.66%)	294 (32.34%)	909 (49.30%)	
Seldom or never	101 (51.01%)	97 (48.99%)	198 (10.74%)	
Unable to answer	10 (37.04%)	17 (62.96%)	27 (1.46%)	
**Feel useless with age**				<0.001
Always	52 (59.77%)	35 (40.23%)	87 (4.72%)	
Often or sometimes	567 (63.85%)	321 (36.15%)	888 (48.16%)	
Seldom or never	641 (77.04%)	191 (22.96%)	832 (45.12%)	
Unable to answer	19 (51.35%)	18 (48.65%)	37 (2.01%)	
**Be happy as younger**				<0.001
Always	93 (68.38%)	43 (31.62%)	136 (7.38%)	
Often or sometimes	762 (76.20%)	238 (23.80%)	1000 (54.23%)	
Seldom or never	407 (60.93%)	261 (39.07%)	668 (36.23%)	
Unable to answer	17 (42.50%)	23 (57.50%)	40 (2.17%)	
**Eat fresh fruit**				0.004
Quite often	541 (72.42%)	206 (27.58%)	747 (40.51%)	
Occasionally	503 (69.57%)	220 (30.43%)	723 (39.21%)	
Rarely or never	235 (62.83%)	139 (37.17%)	374 (20.28%)	
**Eat vegetables**				0.001
Quite often	890 (71.89%)	348 (28.11%)	1238 (67.14%)	

Occasionally	380 (64.74%)	207 (35.26%)	587 (31.83%)	
Rarely or never	9 (47.37%)	10 (52.63%)	19 (1.03%)	
**Kind of grease for cooking**				0.417
Vegetable grease	1066 (69.63%)	465 (30.37%)	1531 (83.03%)	
Gingili grease	2 (50.00%)	2 (50.00%)	4 (0.22%)	
Lard	209 (68.75%)	95 (31.25%)	304 (16.49%)	
Other animal's fat	2 (40.00%)	3 (60.00%)	5 (0.27%)	
**Main flavor**				0.250
Insipidity	707 (67.72%)	337 (32.28%)	1044 (56.62%)	
Salty or sweet	316 (71.66%)	125 (28.34%)	441 (23.92%)	
Hot or crude	52 (76.47%)	16 (23.53%)	68 (3.69%)	
Do not have all	204 (70.10%)	87 (29.90%)	291 (15.78%)	
**Kind of drinking water**				0.356
Boiled water	1210 (69.10%)	541 (30.90%)	1751 (94.96%)	
Un-boiled water	69 (74.19%)	24 (25.81%)	93 (5.04%)	
**Smoke at present**				<0.001
No	939 (66.03%)	483 (33.97%)	1422 (77.11%)	
Yes	340 (80.57%)	82 (19.43%)	422 (22.89%)	
**Drink at present**				<0.001
No	955 (66.27%)	486 (33.73%)	1441 (78.15%)	
Yes	324 (80.40%)	79 (19.60%)	403 (21.85%)	
**Exercise at present**				0.002
No	797 (66.86%)	395 (33.14%)	1192 (64.64%)	
Yes	482 (73.93%)	170 (26.07%)	652 (35.36%)	
**Suffering from hypertension**				0.014
No	999 (71.10%)	406 (28.90%)	1405 (76.19%)	
Yes	254 (63.50%)	146 (36.50%)	400 (21.69%)	
Don't know	26 (66.67%)	13 (33.33%)	39 (2.11%)	
**Suffering from diabetes**				0.220

No	1176 (69.83%)	508 (30.17%)	1684 (91.32%)	
Yes	76 (66.67%)	38 (33.33%)	114 (6.18%)	
Don’t know	27 (58.70%)	19 (41.30%)	46 (2.49%)	
**Suffering from heart disease**				0.674
No	1085 (69.33%)	480 (30.67%)1	1565 (84.87%)	
Yes	173 (70.61%)	72 (29.39%)	245 (43.36%)	
Don’t know	21 (61.76%)	13 (38.24%)	34 (1.84%)	
**Suffering from stroke or cvd**				0.432
No	1151 (69.30%)	510 (30.70%)	1661 (90.08%)	
Yes	106 (72.11%)	41 (27.89%)	147 (7.97%)	
Don’t know	22 (61.11%)	14 (38.89%)	36 (1.95%)	
**Suffering from arthritis**				0.402
No	1099 (69.96%)	472 (30.04%)	1571 (85.20%)	
Yes	157 (65.69%)	82 (34.31%)	239 (12.96%)	
Don’t know	23 (67.65%)	11 (32.35%)	34 (1.84%)	
**Household income (Numeric)**				0.948
Mean: 34145.620, Std: 31662.079, Min: 0, Max: 99999.0				
**Years of schooling**				<0.001
Illiterate	549 (60.13%)	364 (39.87%)	913 (49.51%)	
Primary school stage	524 (76.50%)	161 (23.50%)	685 (37.15%)	
High school stage	184 (84.40%)	34 (15.60%)	218 (11.82%)	
University and above	22 (78.57%)	6 (21.43%)	28 (1.52%)	
**Occupation before retirement**				<0.001
Knowledge /management practitioner	122 (84.14%)	23 (15.86%)	145 (7.86%)	
Front-line operation /service practitioner	1056 (68.75%)	480 (31.25%)	1536 (83.30%)	
Autonomous/non-employed practitioners	67 (58.77%)	47 (41.23%)	114 (6.18%)	
Special/non-employment groups	34 (69.39%)	15 (30.61%)	49 (2.66%)	

Notes: BADL, basic activities of daily living; IADL, instrumental activities of daily living. P values were calculated using t-tests for continuous variables (e.g., ABSIzScore) and chi-square tests for categorical variables (e.g., Sex, Marriage). For the association between ABSIzScore and anxiety symptoms: male trend line slope = 0.03 (95% CI: 0.01–0.05), female

slope $=0.02$ (95% CI: 0.003–0.037); inter-sex slope difference: $z=2.14$, $p=0.032$ (linear regression with sex×ABSIzScore interaction term). 95% CIs for anxiety prevalence in key subgroups: age ⩾ 85 years =70.97% (64.21% – 77.73%), divorced/widowed =45.48% (41.65% – 49.31%), illiterate =39.87% (36.65% – 43.09%).
