# Supplementary material for: Interpretable ensemble learning model with shapley additive explanations for predicting anxiety symptoms risk in Chinese older adults with body shape index abnormality
Source: PLoS One. 2025 Oct 30;20(10):e0335437. doi: 10.1371/journal.pone.0335437 (PMC12574866; doi:10.1371/journal.pone.0335437)
Supplement: S4 Table — (PDF) [file pone.0335437.s004.pdf]

Table S4

Performance of predicton models for three balancing methods on three ensemble leanings in internal test set

Model	AUC	CA	F1	Prec	Recall	MCC
Boosting-SET-internal test	0.806	0.789	0.783	0.782	0.789	0.484
Stacking-SET-internal test	0.783	0.751	0.748	0.746	0.751	0.404
Voting-SET-internal test	0.733	0.776	0.774	0.772	0.776	0.466
Boosting-ADASYN-internal test	0.814	0.794	0.786	0.786	0.794	0.493
Stacking-ADASYN-internal test	0.755	0.739	0.740	0.741	0.739	0.392
Voting-ADASYN-internal test	0.739	0.786	0.784	0.783	0.786	0.490
Boosting-BS-internal test	0.810	0.796	0.791	0.790	0.796	0.503
Stacking-BS-internal test	0.777	0.759	0.756	0.754	0.759	0.423
Voting-BS-internal test	0.722	0.769	0.765	0.763	0.769	0.443

Note: AUC: area under the curve; CA: classification accuracy; F1: F1–score; Prec: precision; MCC: matthews correlation coefficient; SMOTE-ENN+Tomek: Synthetic Minority Oversampling Technique, Edited Nearest Neighbors and Tomek Links; ADASYN: Adaptive Synthetic Sampling; BS: BorderlineSMOTE. Base learners include single-algorithm models (LR, kNN, DT, Gaussian Naïve Bayes [Gaussian NB, alpha=0.1], SVM, NN, SGD Classifier) and composite ensemble models (RF, XGBoost). 95% CIs for all performance metrics and statistical comparisons between ensemble models are reported in Figure 5's note.
