# Supplementary material for: Interpretable ensemble learning model with shapley additive explanations for predicting anxiety symptoms risk in Chinese older adults with body shape index abnormality
Source: PLoS One. 2025 Oct 30;20(10):e0335437. doi: 10.1371/journal.pone.0335437 (PMC12574866; doi:10.1371/journal.pone.0335437)
Supplement: S5 Table — (PDF) [file pone.0335437.s005.pdf]

Table S5

Performance of predicton models for three balancing methods on three ensemble leanings in external test set in 2011

Model	AUC	CA	F1	Prec	Recall	MCC
Boosting-SET-2011	0.769	0.756	0.755	0.635	0.792	0.397
Stacking -SET-2011	0.735	0.726	0.756	0.756	0.756	0.359
Voting-SET-2011	0.719	0.741	0.719	0.709	0.752	0.354
Boosting-ADASYN-2011	0.772	0.754	0.744	0.734	0.776	0.387
Stacking-ADASYN-2011	0.707	0.709	0.775	0.735	0.727	0.281
Voting-ADASYN-2011	0.724	0.734	0.757	0.716	0.763	0.314
Boosting-BS-2011	0.756	0.731	0.705	0.785	0.744	0.331
Stacking-BS-2011	0.695	0.697	0.782	0.709	0.760	0.270
Voting-BS-2011	0.715	0.719	0.749	0.768	0.771	0.282

Note: AUC: area under the curve; CA: classification accuracy; F1: F1 – score; Prec: precision; MCC: matthews correlation coefficient; SMOTE-ENN+Tomek: Synthetic Minority Oversampling Technique, Edited Nearest Neighbors and Tomek Links; ADASYN: Adaptive Synthetic Sampling; BS: BorderlineSMOTE.. Base learners include single-algorithm models (LR, kNN, DT, Gaussian Naïve Bayes [Gaussian NB, alpha=0.1], SVM, NN, SGD Classifier) and composite ensemble models (RF, XGBoost). 95% CIs for all performance metrics and statistical comparisons between ensemble models are reported in Figure 5's note.
