# Supplementary material for: Interpretable ensemble learning model with shapley additive explanations for predicting anxiety symptoms risk in Chinese older adults with body shape index abnormality
Source: PLoS One. 2025 Oct 30;20(10):e0335437. doi: 10.1371/journal.pone.0335437 (PMC12574866; doi:10.1371/journal.pone.0335437)
Supplement: S6 Table — (PDF) [file pone.0335437.s006.pdf]

Table S6

Performance of predicton models for three balancing methods on three ensemble leanings in external test set in 2014

Model	AUC	CA	F1	Prec	Recall	MCC
Boosting-SET-2014	0.777	0.746	0.735	0.734	0.746	0.369
Stacking -SET-2014	0.697	0.701	0.695	0.692	0.701	0.276
Voting-SET-2014	0.730	0.724	0.691	0.702	0.724	0.276
Boosting-ADASYN-2014	0.766	0.754	0.743	0.742	0.754	0.387
Stacking-ADASYN-2014	0.698	0.709	0.699	0.695	0.709	0.281
Voting-ADASYN-2014	0.706	0.734	0.711	0.716	0.734	0.314
Boosting-BS-2014	0.753	0.731	0.720	0.717	0.731	0.331
Stacking-BS-2014	0.695	0.697	0.692	0.689	0.697	0.270
Voting-BS-2014	0.694	0.719	0.700	0.699	0.719	0.282

Note: AUC: area under the curve; CA: classification accuracy; F1: F1 – score; Prec: precision; MCC: matthews correlation coefficient; SMOTE-ENN+Tomek: Synthetic Minority Oversampling Technique, Edited Nearest Neighbors and Tomek Links; ADASYN: Adaptive Synthetic Sampling; BS: BorderlineSMOTE. Base learners include single-algorithm models (LR, kNN, DT, Gaussian Naïve Bayes [Gaussian NB, alpha=0.1], SVM, NN, SGD Classifier) and composite ensemble models (RF, XGBoost). 95% CIs for all performance metrics and statistical comparisons between ensemble models are reported in Figure 5's note.
