# Supplementary figures and images for: Interpretable ensemble learning model with shapley additive explanations for predicting anxiety symptoms risk in Chinese older adults with body shape index abnormality
Source: PLoS One. 2025 Oct 30;20(10):e0335437. doi: 10.1371/journal.pone.0335437 (PMC12574866; doi:10.1371/journal.pone.0335437)

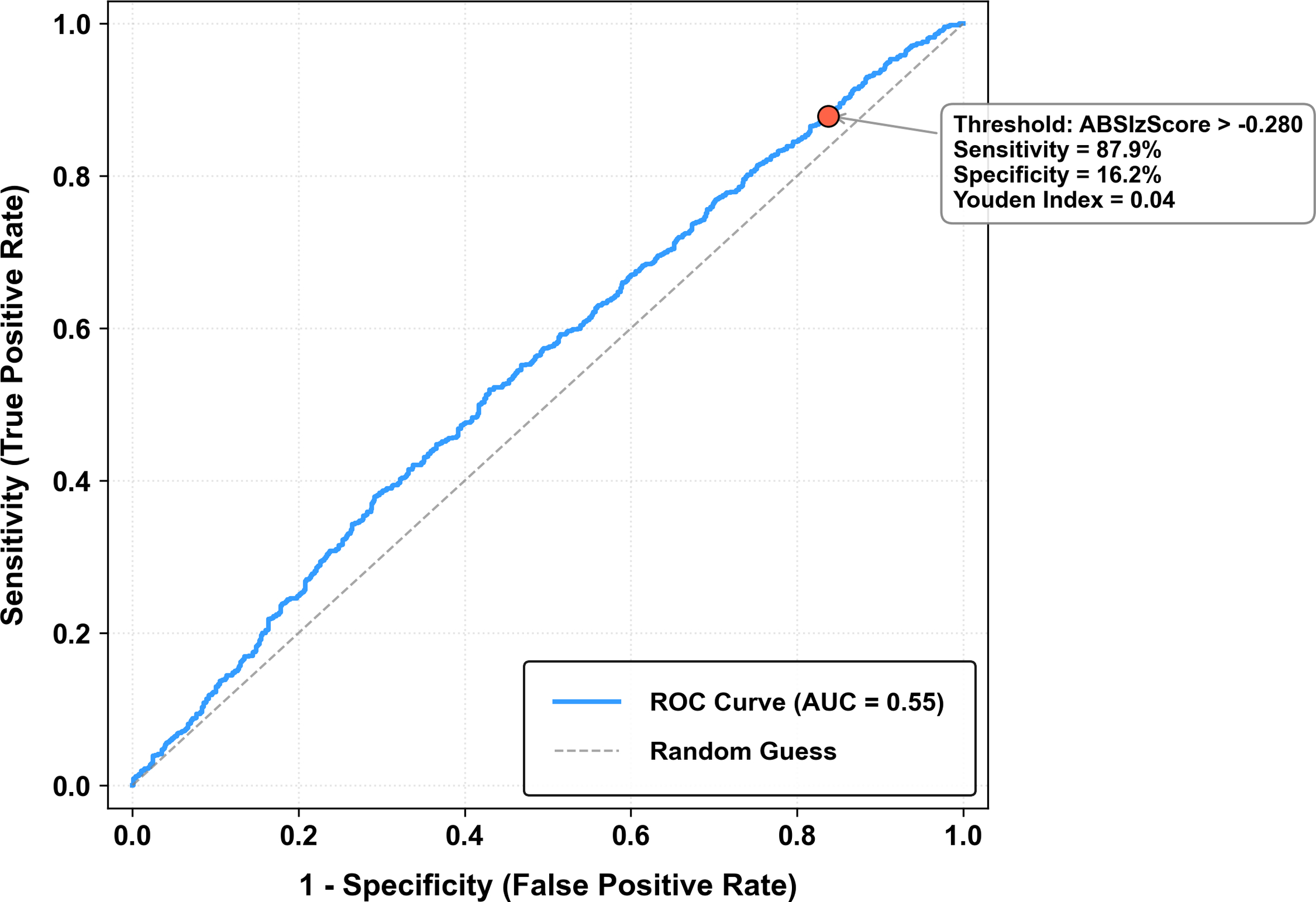

Supplement: S1 Fig — (TIF) [file pone.0335437.s007.tif]

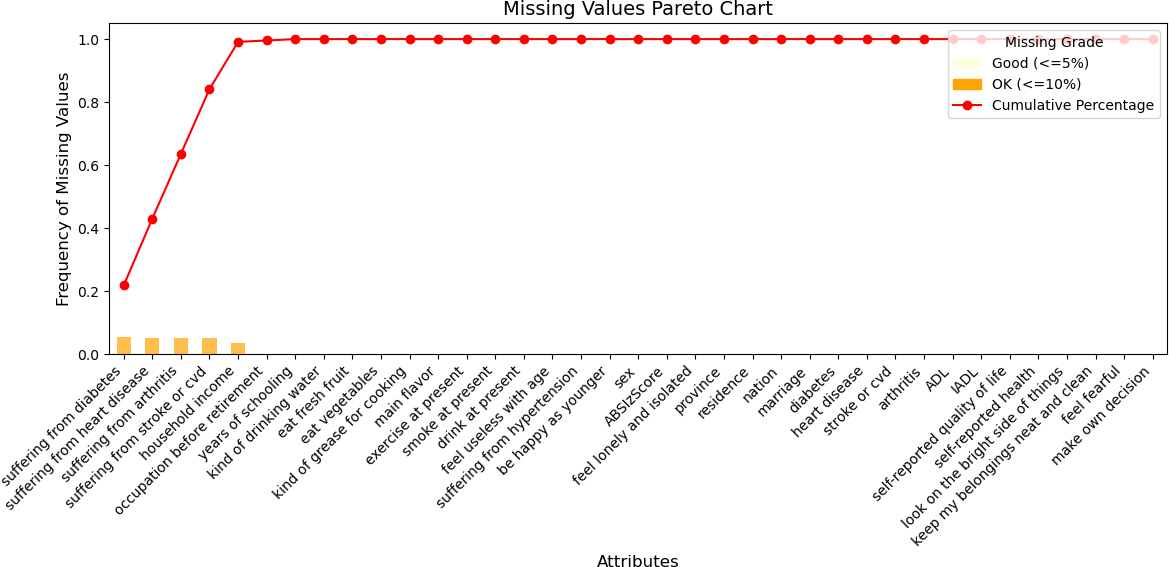

Supplement: S2 Fig — (TIF) [file pone.0335437.s008.tif]

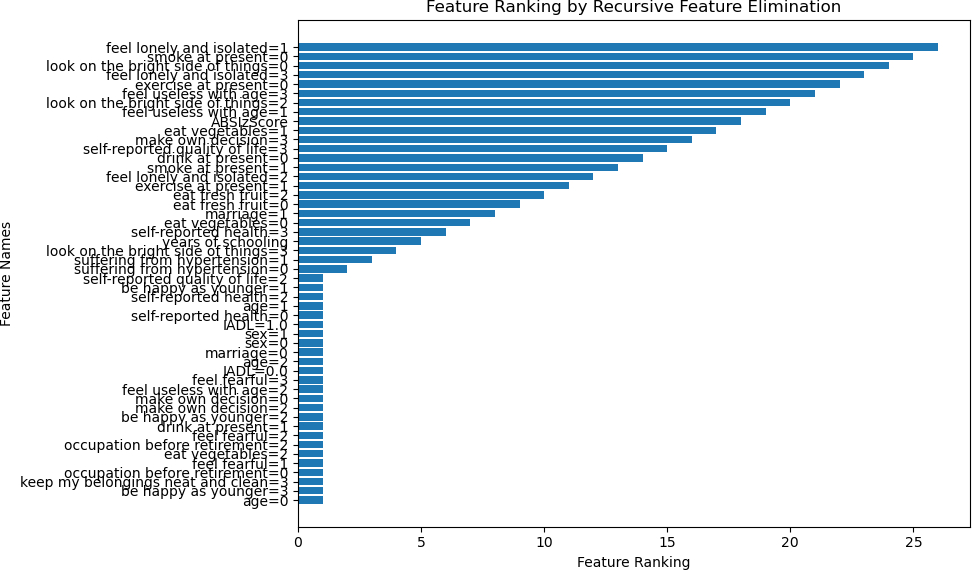

Supplement: S3 Fig — (TIF) [file pone.0335437.s009.tif]

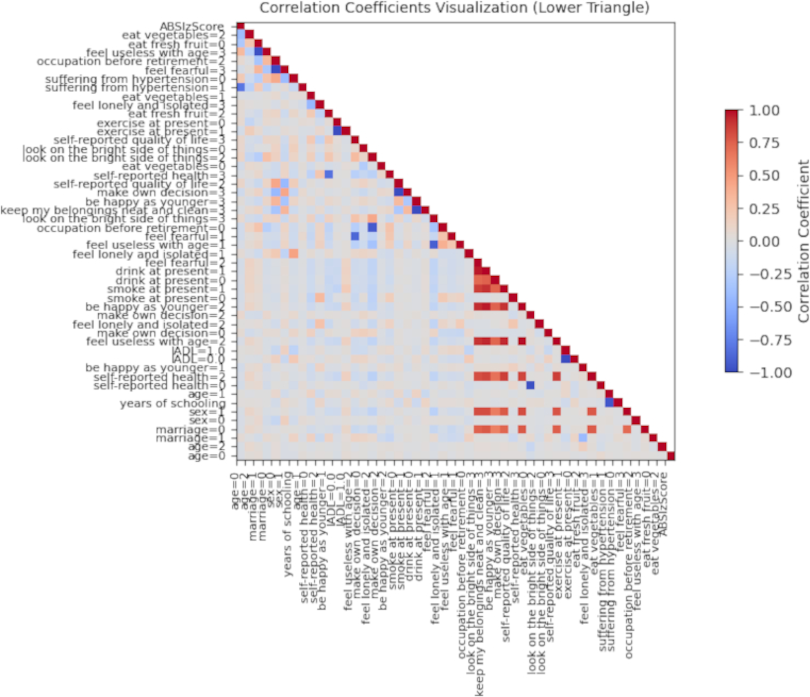

Supplement: S4 Fig — (TIF) [file pone.0335437.s010.tif]

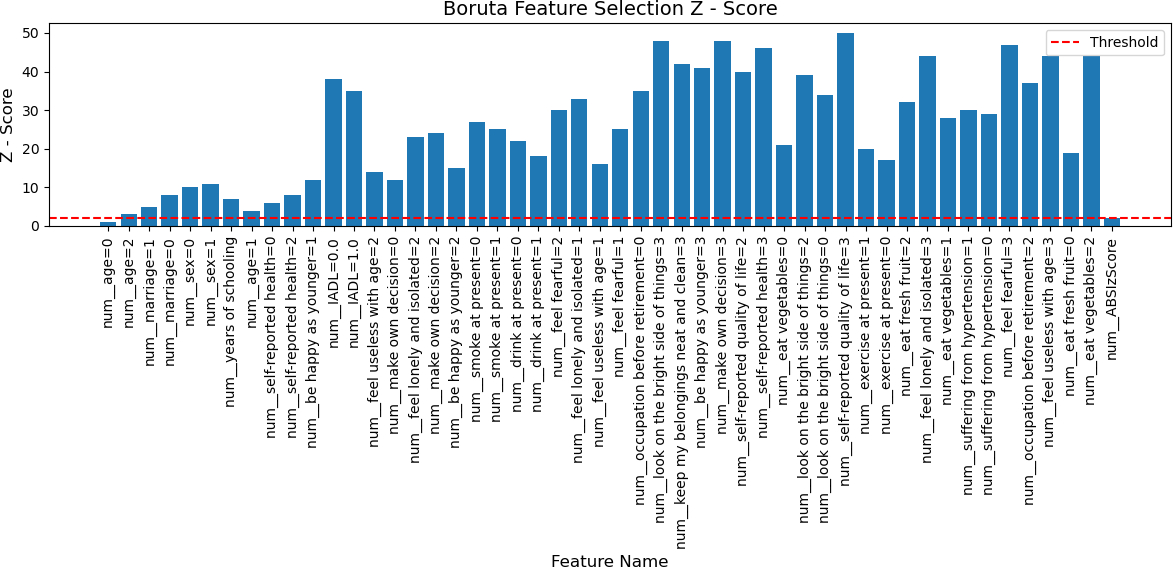

Supplement: S5 Fig — (TIF) [file pone.0335437.s011.tif]

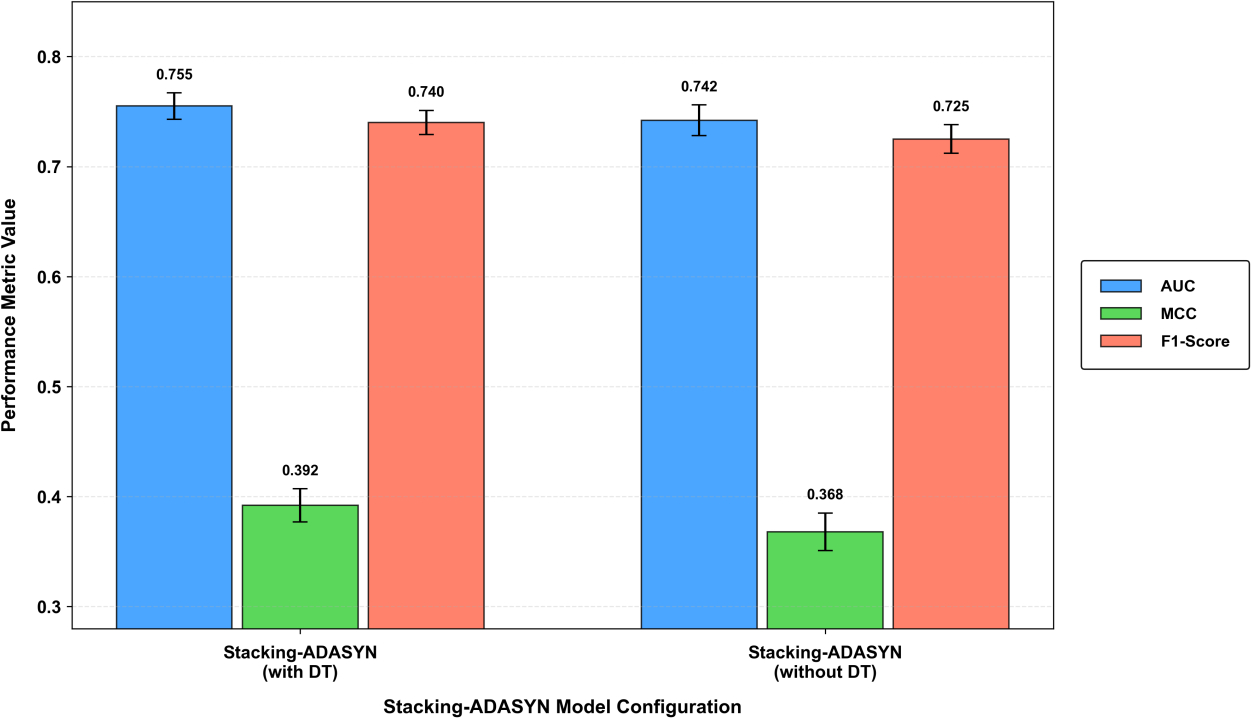

Supplement: S6 Fig — (TIF) [file pone.0335437.s012.tif]
